# *O*-GlcNAcylation: an important post-translational modification and a potential therapeutic target for cancer therapy

**DOI:** 10.1186/s10020-022-00544-y

**Published:** 2022-09-14

**Authors:** Qingsong Lu, Xiaozhen Zhang, Tingbo Liang, Xueli Bai

**Affiliations:** 1 Department of Hepatobiliary and Pancreatic Surgery, The First Affiliated Hospital, School of Medicine, Zhejiang University, Hangzhou, Zhejiang China; 2 Zhejiang Provincial Key Laboratory of Pancreatic Disease, The First Affiliated Hospital, School of Medicine, Zhejiang University, Hangzhou, Zhejiang China; 3 Zhejiang Provincial Innovation Center for the Study of Pancreatic Diseases, Zhejiang University, Hangzhou, Zhejiang China; 4 Zhejiang Provincial Clinical Research Center for the Study of Hepatobiliary and Pancreatic Diseases, Zhejiang University, Hangzhou, China; 5 Cancer Center, Zhejiang University, Hangzhou, China; 6 Research Center for Healthcare Data Science, Zhejiang Lab, Hangzhou, Zhejiang China

**Keywords:** *O*-GlcNAc, Post-translational modification, OGT, OGA, Biomarker, Cancer therapy

## Abstract

*O*-linked β-d-*N*-acetylglucosamine (*O*-GlcNAc) is an important post-translational modification of serine or threonine residues on thousands of proteins in the nucleus and cytoplasm of all animals and plants. In eukaryotes, only two conserved enzymes are involved in this process. *O*-GlcNAc transferase is responsible for adding *O*-GlcNAc to proteins, while *O*-GlcNAcase is responsible for removing it. Aberrant *O*-GlcNAcylation is associated with a variety of human diseases, such as diabetes, cancer, neurodegenerative diseases, and cardiovascular diseases. Numerous studies have confirmed that *O*-GlcNAcylation is involved in the occurrence and progression of cancers in multiple systems throughout the body. It is also involved in regulating multiple cancer hallmarks, such as metabolic reprogramming, proliferation, invasion, metastasis, and angiogenesis. In this review, we first describe the process of *O*-GlcNAcylation and the structure and function of *O*-GlcNAc cycling enzymes. In addition, we detail the occurrence of *O*-GlcNAc in various cancers and the role it plays. Finally, we discuss the potential of *O*-GlcNAc as a promising biomarker and novel therapeutic target for cancer diagnosis, treatment, and prognosis.

## Background

*O*-linked β-d-*N*-acetylglucosamine (*O*-GlcNAc) is an important post-translational modification (PTM) comprising the reversible, highly dynamic, covalent attachment of β-*N*-GlcNAc to Ser/Thr residues on proteins, which was first reported by Hart in 1984 (Torres and Hart 1984). Unlike conventional complex glycans decorating the surface of cells, *O*-GlcNAc is a simple monosaccharide modification that mostly occurs inside cells, specifically in the nucleus or cytoplasm. With the development of technologies related to identification, site mapping, quantitation, and site-specific *O*-GlcNAc protein function determination of *O*-GlcNAc proteins (Ma and Hart 2014), studies have identified more than 16,000 proteins that are *O*-GlcNAcylated in 42 species (Wulff-Fuentes et al. 2021), including cytoskeletal proteins and their regulatory proteins (Arnold et al. 1996; Ding and Vandre 1996; Takahashi et al. 1999), nucleoporins (Davis and Blobel 1987), synaptic proteins (Luthi et al. 1991), heat shock proteins (Roquemore et al. 1996), tumor suppressor proteins, RNA polymerase II catalytic subunit (Kelly et al. 1993; Cervoni et al. 1997), as well as multiple transcription factors (Jackson and Tjian 1988; Yang et al. 2001; Iyer et al. 2003). These proteins are involved in all aspects of cellular function, including metabolism, signal transduction, transcriptional regulation, cell cycle control, protein trafficking, and regulation of the cell structure (Wells et al. 2001; Love and Hanover 2005; Zachara and Hart 2006; Slawson and Hart 2011).

*O*-GlcNAcylation has many unique characteristics compared to "classical" glycosylation. Chemically, *O*-GlcNAcylation only adds monosaccharides to the Ser/Thr residues of proteins and does not involve complex glycan structures. Spatially, *O*-GlcNAcylation mainly targets proteins in the nucleus, cytoplasm, and mitochondria rather than on the cell membrane (Hu et al. 2009). Temporally, similar to phosphorylation/dephosphorylation, *O*-GlcNAcylation achieves highly dynamic and fast-cycling PTM by reversibly adding and removing β-N-GlcNAc on protein Ser/Thr residues.

The diversity and importance of *O*-GlcNAc-modified proteins mean that a variety of human disorders are linked to aberrant *O*-GlcNAc, including diabetes (Issad et al. 2010; Ma and Hart 2013) and diabetic complications (Peterson and Hart 2016; Degrell et al. 2009; Akimoto et al. 2011), cardiovascular diseases (Chatham et al. 2008; Laczy et al. 2009; Fulop et al. 2007; Clark et al. 2003; Ngoh et al. 2009), cancer (Slawson and Hart 2011; Ma and Vosseller 2013; Fardini et al. 2013), neurodegenerative diseases (such as Alzheimer's disease) (Lazarus et al. 2009; Gong et al. 2012; Zhu et al. 2014), and immune system disorders (Golks and Guerini 2008; Golks et al. 2007). In recent years, because of the large proportion of cancer deaths among all deaths worldwide (Erratum: Global cancer statistics 2018: GLOBOCAN estimates of incidence and mortality worldwide for 36 cancers in 185 countries. 2018), increasing numbers of studies have focused on the role of *O*-GlcNAcylation in many cancers, including their metabolism, proliferation, angiogenesis, and metastasis (Ferrer et al. 2016; Wu et al. 2020; Ma and Vosseller 2014). In this review, we first describe the general mechanism of *O*-GlcNAcylation and the structure of *O*-GlcNAc regulating enzymes and then focus on the important role played by *O*-GlcNAcylation in various cancers. Finally, we discuss the potential of *O*-GlcNAc as a cancer diagnostic and prognostic biomarker and therapeutic target.

## The basic process of *O*-GlcNAcylation

**Fig. 1:**
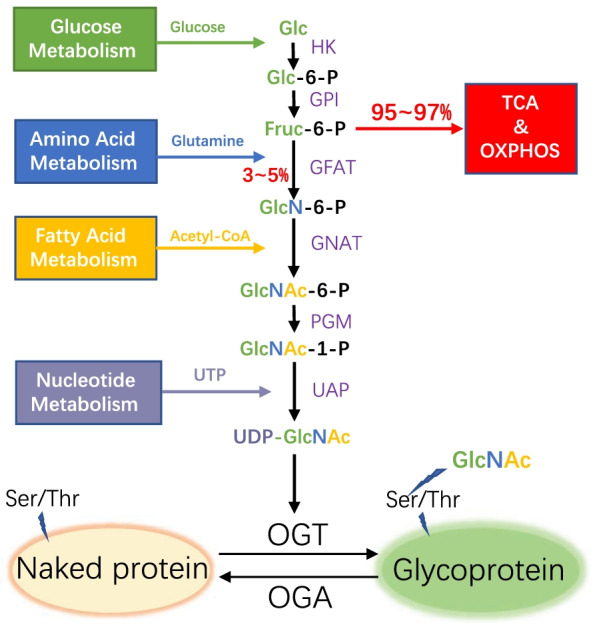
The process of hexosamine biosynthetic pathway (HBP) and *O*-GlcNAcylation. HBP integrates multiple metabolic pathways and the end product UDP-GlcNAc acts as a sugar donor for protein *O*-GlcNAcylation. *HK* hexokinase, *GPI* glucose-6-phosphate isomerase, *GFAT* glutamine:fructose-6-phosphate amidotransferase, *GNAT* glucosamine 6-phosphate *N*-acetyltransferase, *PGM* phosphoglucomutase, *UAP* UDP-*N*-acetylglucosamine pyrophosphorylase, *TCA* tricarboxylic acid cycle, *OXPHOS* oxidative phosphorylation

Under euglycemic conditions, most glucose taken up by cells enters the glycolytic metabolic pathway; however, 3–5% of the glucose is still separated from the glycolytic pathway into the hexosamine biosynthetic pathway (HBP) (Marshall et al. 1991). In this process, an intermediate of glycolysis, fructose-6-phosphate, is converted to glucosamine 6-phosphate, catalyzed by glutamine fructose-6-phosphate amidotransferase (GFAT), and then the end product of the HBP, UDP-GlcNAc, is synthesized in the presence of many different metabolites, such as the fatty acid metabolite acetyl-coA and the nucleic acid metabolite UTP (Dennis et al. 2009). Eventually, UDP-GlcNAc acts as the sugar donor for *O*-GlcNAc. Under the action of *O*-GlcNAc modifying enzymes, β-*N*-GlcNAc is provided to complete the dynamic modification of countless intracellular proteins (Fig. 1).

### *O*-GlcNAc modifying enzymes

As an extensive PTM, the number of proteins modified by *O*-GlcNAc is similar to that of phosphorylation (Bond and Hanover 2015). However, unlike phosphorylation/dephosphorylation, only two highly conserved enzymes are involved in the addition and removal of *O*-GlcNAc (Hart 2019; Kreppel et al. 1997; Lubas et al. 1997). *O*-GlcNAc transferase (OGT) is responsible for connecting β-*N*-GlcNAc to the substrate protein, and β-N-acetylglucosaminidase (*O*-GlcNAcase, OGA) removes it. In contrast to this, there are more than 1000 kinases and more than 500 phosphatases involved in phosphorylation/dephosphorylation (Cohen 2000).

#### *O*-GlcNAc transferase (OGT)

**Fig. 2:**
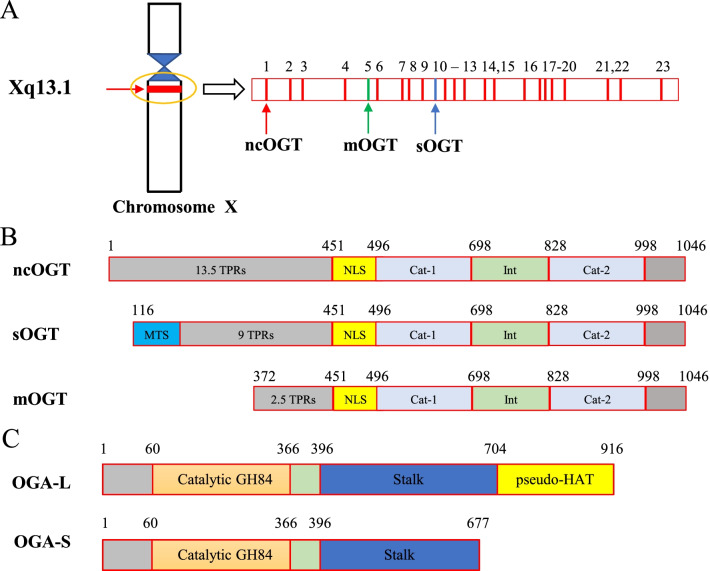
Schematic diagram of the gene and protein structure of *O*-GlcNAc modifying enzymes. Human OGT is composed of three regions and three splice forms are known. An *N*-terminal domain is formed from a series of TPRs and a *C*-terminal domain contains the glycosyltransferase catalytic domain. **A** Schematic of the genomic structure of the human OGT splice variants. **B** The linear domain organization is shown for the three OGT isoforms with the amino acids marking the boundary of each domain indicated. The TPRs are shown in light gray, the nuclear localization sequence (NLS) is in yellow, and the glycosyltransferase catalytic regions are in light blue (Cat-1and Cat-2). **C** The linear domain organization is shown for the two OGA isoforms with the amino acids marking the boundary of each domain indicated. The Catalytic GH84 is shown in orange, the pseudo-HAT is shown in yellow

OGT is the only enzyme responsible for the addition of *O*-GlcNAc to the Ser/Thr residues of target proteins. In humans, the gene encoding OGT is located near the centromere of the X chromosome (Xq13.1) (Nolte and Muller 2002) and contains 23 exons and 21 introns. As a result of alternative splicing, there are three independent isoforms with different amino termini: nucleocytoplasmic OGT (ncOGT), mitochondrial OGT (mOGT), and short form OGT (sOGT) (Nolte and Muller 2002). Among them, ncOGT is the longest isoform and comprises 1046 amino acids, encoded by exons 1–4 and 6–23 (Hanover et al. 2003). The second longest, mOGT, is encoded by exons 5–23, and comprises 931 amino acids. mOGT localizes to the mitochondria, which is determined by its particular mitochondrial targeting sequence (MTS) at the N-terminus (Love et al. 2003). The shortest isoform, sOGT, is encoded by exons 10–23 and comprises 675 amino acids (Hanover et al. 2003) (Fig. 2A). OGT is widely expressed in all tissues, but varies in content, with the highest expression in pancreatic β-cells and brain, and the lowest in the liver and lung. (Weinstein et al. 2013; Alonso et al. 2014; Vander et al. 2009)

From the crystal structure, OGT is composed of three regions: a tripeptide repeat (TPR) motif at the amino (N) terminal domain, a catalytic domain at the carboxyl (C) terminus, and a nuclear localization sequence (NLS) connecting these two domains (Kreppel et al. 1997; Lubas et al. 1997). The three isomers share the same C-terminal catalytic domain, which comprises an insertion domain separating two catalytic sheets. However, there is a structural difference at the N-terminus, which is manifested in the different number of TPR motifs: ncOGT contains 13.5 TPRs, mOGT contains 9, while sOGT contains only 2.5 TPRs (Ju 2020) (Fig. 2B).

Given that dysregulation of *O*-GlcNAcylation is associated with various diseases, tuning of OGT function is necessary and is achieved in three ways: First, the activity of OGT is influenced by the concentration of metabolites and enzymes involved in HBP, especially the precise regulation of the end product UDP-GlcNAc (Kreppel and Hart 1999); second, OGT itself is modified by various PTMs, such as phosphorylation, ubiquitination, (Peng et al. 2021) and S-Nitration (Ryu and Do 2011). Phosphorylation at Thr444 of OGT by AMP-activated protein kinase (AMPK) can alter its substrate selectivity and nuclear localization (Bullen et al. 2014); and glycogen synthase kinase-3β (GSK3β) can activate OGT by phosphorylating serine residues on OGT and regulates circadian rhythms (Kaasik et al. 2013). Interestingly, OGT can be *O*-GlcNAcylated (Fan et al. 2018; Griffin et al. 2016; Tai et al. 2004): *O*-GlcNAc at Ser389 affects its nuclear localization, whereas *O*-GlcNAc at Thr12 and Ser56 alters its substrate selectivity (Seo et al. 2016; Liu et al. 2019a). Finally, OGT can interact with other proteins thereby regulating its activity: under glucose deprivation, OGT can influence the activity of specific proteins by interacting with the stress kinase p38 (Cheung and Hart 2008); and peroxisome proliferator-activated receptor gamma coactivator 1-alpha (PGC-1α), a transcriptional coactivator under hyperglycemic conditions, interacts with OGT to promote the activity of OGT on the transcription factor forkhead box O1 (FOXO1) (Housley et al. 2008, 2009).

**Fig. 3:**
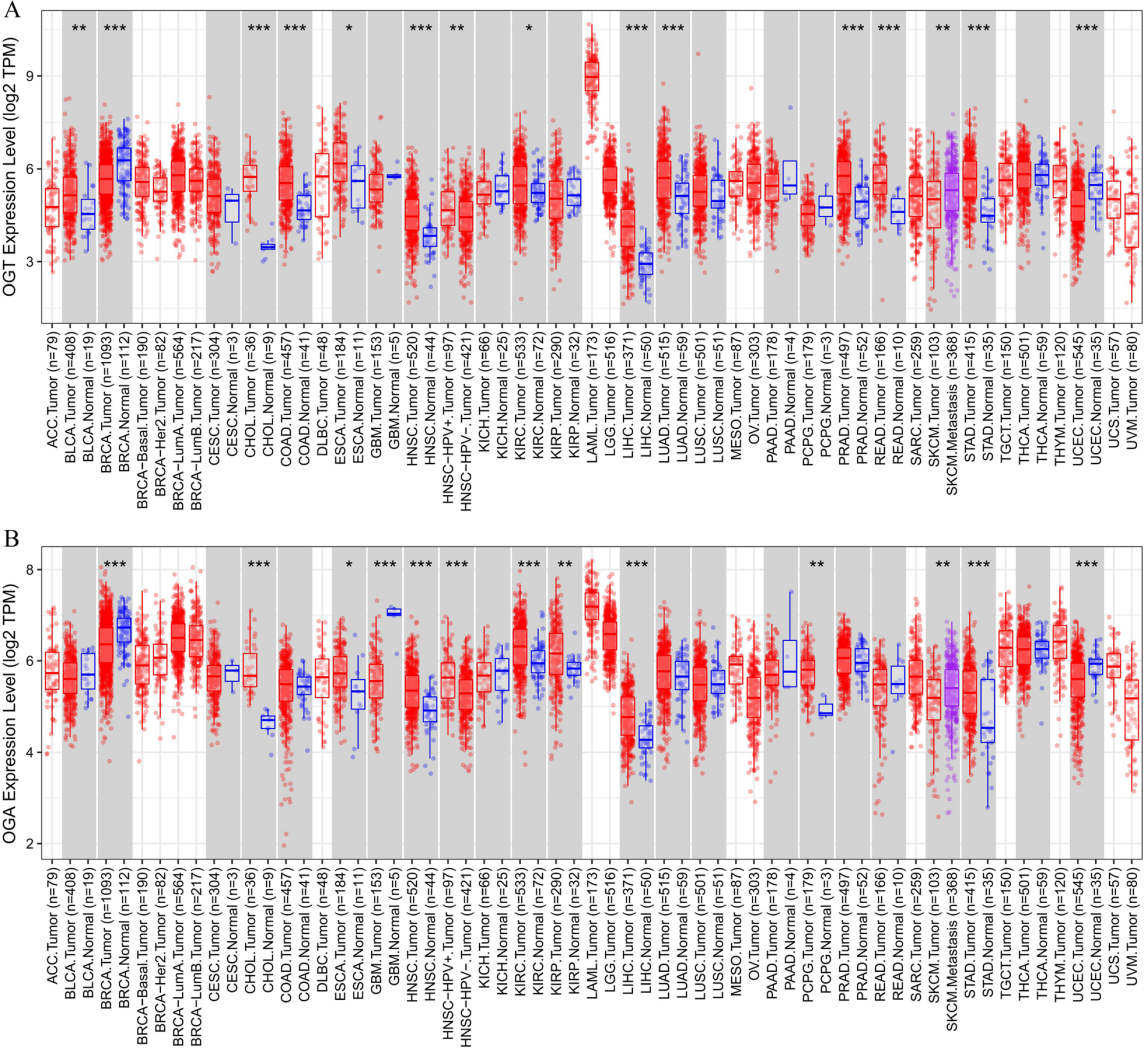
Expression profile analyses of *O*-GlcNAc modifying enzymes across multiple cancers and normal tissues. Expression pattern of *OGT *(**A**) and *OGA* (**B**) in ACC, BLCA, BRCA, CESC, CHOL, COAD, DLBC, ESCA, GBM, HNSC, KICH, KIRC, KIRP, LAML, LGG, LIHC, LUAD, LUSC, OV, PAAD, PCPG, PRAD, READ, SARC, SKCM, STAD, TGCT, THCA, THYM, UCEC, and UCS. TIMER2.0 was used to generate box plots profiling *O*-GlcNAc modifying enzymes expression patterns across multiple cancer types (TCGA tumor) and adjacent normal tissue samples (TCGA normal). Each dot represents the individual expression of a distinct tumor or normal sample. The statistical significance computed by the Wilcoxon test is annotated by the number of stars (*p-value < 0.05; **p-value < 0.01; ***p-value < 0.001). As displayed in gray columns when normal data is available

Abnormal OGT activity affects many downstream proteins. Therefore, the expression of OGT often changes in diseases states, especially in various cancers. To further understand the potential roles and clinical relevance of OGT in human cancers, our group investigated the *OGT* expression profiles in 33 major human cancer types in The Cancer Genome Atlas (TCGA, http://cancergenome.nih.gov) database (Weinstein et al. 2013; Blum et al. 2018; Roychowdhury and Chinnaiyan 2016), using Gene_DE module of the Tumor Immune Estimation Resource package (Li et al. 2020) (TIMER2.0, http://timer.cistrome.org). The results showed that: (1) Compared with that in adjacent normal tissues, *OGT* was expressed at markedly higher levels in bladder urothelial carcinoma (BLCA), cholangiocarcinoma (CHOL), colon adenocarcinoma (COAD), esophageal carcinoma (ESCA), head and neck squamous cell carcinoma (HNSC), kidney renal clear cell carcinoma (KIRC), liver hepatocellular carcinoma (LIHC), lung adenocarcinoma (LUAD), prostate adenocarcinoma (PRAD), rectum adenocarcinoma (READ), stomach adenocarcinoma (STAD). and (2) the expression level of *OGT* in breast invasive carcinoma (BRCA) and uterine corpus endometrial carcinoma (UCEC) was significantly lower than that in normal tissues (Fig. 3A).

**Fig. 4:**
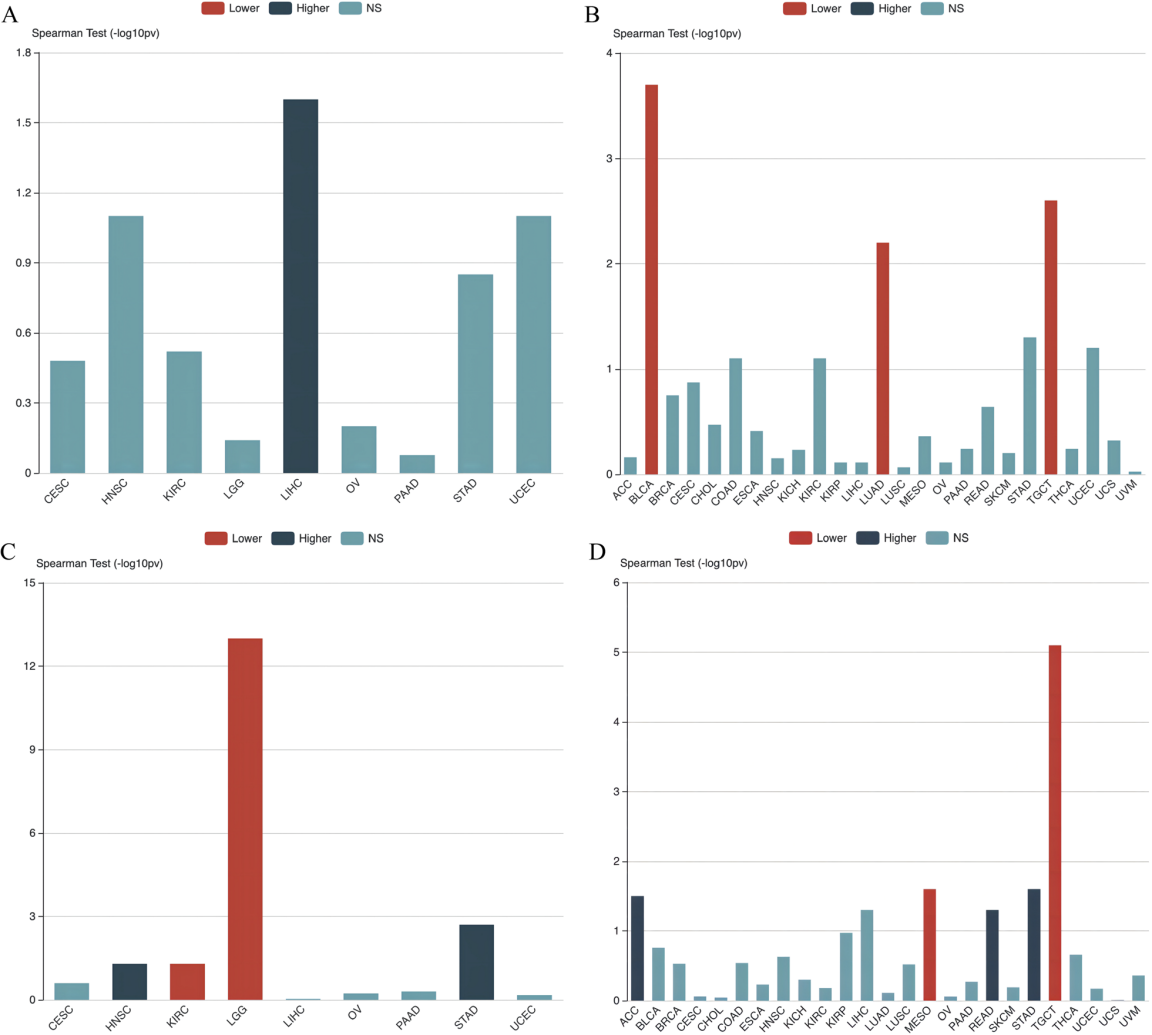
Association analyses between *O*-GlcNAc modifying enzymes and clinical features across multiple cancers. **A** Association between *OGT* expression and grade across human cancers. **B** Association between *OGT* expression and stage across human cancers. **C** Association between *OGA* expression and grade across human cancers. **D** Association between *OGA* expression and stage across human cancers. TISIDB was used to generate associations between the expression of *OGT* and *OGA* and pathological distribution across multiple cancers (TCGA tumor). Spearman test was used to calculate the associations, and a p-value < 0.05 was considered statistically significant

Furthermore, we analyzed the association between *OGT* expression and tumor-node-metastasis (TNM) stage, and the tumor grades across 33 cancer types using the Tumor and Immune System Interaction Database (TISIDB, http://cis.hku.hk/TISIDB) (Ru et al. (2019). The results showed that across 33 cancer types: (1) *OGT* expression was associated with higher grades only of LIHC (Fig. 4A); and (2) *OGT* expression was associated with lower tumor stages in BLCA, testicular germ cell tumors (TGCT), and LUAD (Fig. 4B) Overall, the expression of *OGT* is diverse in various cancers and is closely related to the grade and stage of tumors.

#### *O*-GlcNAcase (OGA)

*O*-GlcNAcase (OGA) is only enzyme which removes *O*-GlcNAc from the target proteins. In humans, OGA is encoded by the *OGA* gene on chromosome 10 (10q24.1–q24.3) and maps to a genetic loci involved in Alzheimer’s disease (Bertram et al. 2000; Kuwano et al. 2006). Human *OGA* contains 17 exons and encodes at least two (and possibly more) OGA isoforms of different lengths, caused by alternative splicing of the mRNA. To date, long form OGA (OGA-L) and short form OGA (OGA-S) have been well studied. OGA-L is the longest of the isomers and consists of 916 amino acids. OGA-S is a shorter isomer consisting of only 677 amino acids (Vocadlo 2012). OGA is a dual-domain protein, its N-terminus contains a member of the glycoside hydrolase family 84 (GH84) domain and is considered the catalytic domain (Hanover 2001) (Fig. 2C). The C-terminal domain is believed to have histone acetyltransferase (HAT) activity (Toleman et al. 2004), although this conclusion is still a matter of debate (Butkinaree et al. 2008; Alonso et al. 2014; Schultz and Pils 2002). Between the GH84 and HAT domains, there is an unstructured region in which Asp413 contains a cleavage site for caspase-3 and is processed during apoptosis (Butkinaree et al. 2008). Like OGT, OGA is expressed in all tissues, but varies in content (Gao et al. 2001). The highest expression is observed in the brain, lymph nodes, and spleen, while the lowest expression is observed in the pancreas and salivary glands.

Long-term difficulties in determining the OGA structure mean that the regulatory mechanism of OGA remains to be investigated. OGA is known to be modified by PTMs such as *O*-GlcNAcylation (Woo et al. 2018), phosphorylation (Dephoure et al. 2008; Olsen et al. 2010), and ubiquitination (Akimov et al. 2018). For example, OGA itself can be *O*-GlcNAcylated at Ser398, 399, 405, 410, and Thr415 as a substrate of OGT; however, the mechanism by which these PTMs regulate the function of OGA is largely unknown.

Similar to OGT, we used TCGA, TIMER2.0, and TISIDB to investigate the prognostic influence, pathological features, and clinical relevance of OGA expression in 33 major human cancer types (Weinstein et al. 2013; Blum et al. 2018; Roychowdhury and Chinnaiyan 2016). The results showed that, compared with that in adjacent normal tissues, (1) *OGA* expression is significantly higher in CHOL, ESCA, HNSC, KIRC, kidney renal papillary cell carcinoma (KIRP), LIHC, pheochromocytoma and paraganglioma (PCPG) and STAD. (Fig. 3B). (2) *OGA* expression was associated with higher grades of KIRC and brain lower grade glioma (LGG), but with lower grades of HNSC and STAD (Fig. 4C). (3) *OGA* expression was associated with higher tumor stages in mesothelioma (MESO) and TGCT, but with lower stages in ACC, READ, and STAD (Fig. 4D).

**Fig. 5:**
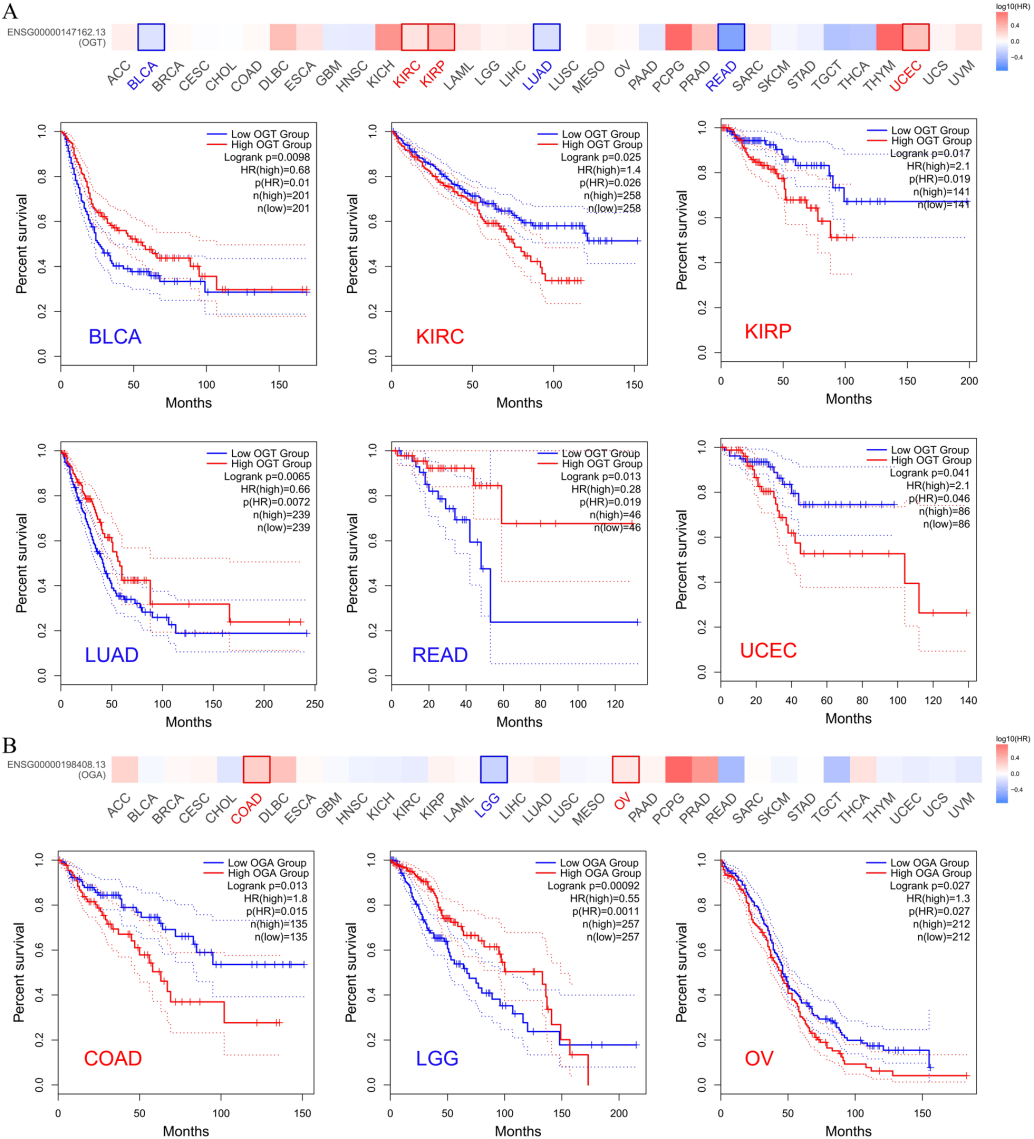
Correlation between *OGT* and *OGA* expression and survival prognosis of cancers in TCGA. We used the GEPIA2 tool to perform overall survival analyses of different tumors in TCGA by *OGT* (**A**) and *OGA* (**B**) expression. The survival map and Kaplan–Meier curves with positive results are given

In addition, we further analyzed the relationship between the overall survival (OS) and the expression of OGT and OGA in 33 major human cancer types using the Gene Expression Profiling Interactive Analysis (GEPIA2, http://gepia2.cancer-pku.cn) package (Tang et al. 2019), the results showed that: (1) *OGT* expression was related to the OS of BLCA, KIRC, KIRP, LUAD, and READ, although the trends were inconsistent (Fig. 5A). (2) *OGA* expression was associated with the OS of COAD, LGG, and ovarian serous cystadenocarcinoma (OV), although the trend was inconsistent (Fig. 5B). In conclusion, the expression patterns of *OGT* and *OGA* are diverse and have both positive and negative effects on clinicopathological features, which indicates that the role of *O*-GlcNAc in cancer is very complex.

## *O*-GlcNAc and Cancer

An important characteristic of cancer is genomic instability and mutation (Hanahan and Weinberg 2011). In order to more intuitively understand the extent to which cancer is caused by the direct mutation of the *O*-GlcNAc site at Ser /Thr, we first selected the top ten most common mutated genes in the TCGA Pancancer Atlas Studies provided by cbioportal database (Cerami et al. 2012) (https://www.cbioportal.org/), they were *TP53*, *TIN*, *MUC16*, *PIK3CA*, *CSMD3*, *RYR2*, *LRP1B*, *SYNE1*, *FLG*, *USH2A*. Next, we sorted out their mutations at Ser/Thr, and then found their overlap sites according to the *O*-GlcNAc site information provided by the *O*-GlcNAc database (Wulff-Fuentes et al. 2021) (https://www.oglcnac.mcw.edu/). The results show that: (1) among the top ten most common mutations in TCGA Pancancer Atlas Studies, after excluding those proteins that are not included in the *O*-GlcANc database and have not been reported with the *O*-GlcANc site, there are four proteins whose mutation sites overlapped with the *O*-GlcNAc site, namely *TP53*, *TTN*, *MUC16* and *SYNE1*. (2) Among these overlapping sites, the site S149 of TP53 is annotated as "likely oncogenic" while the role of overlapping sites of other genes is unknown (Table1).

**Table 1:** The overlapping sites of *O*-GlcANc and cancer mutation

Gene	Mutation proportion (%)	Protein	Uniprot ID	*O*-GlcNAc site	Cancer Type	Overlapping site	Protein Change	Annotation
*TP53*	37	P53	P04637	S149	Serous Ovarian Cancer	S149	S149Ffs*32	The TP53 S149Ffs*32 mutation is likely oncogenic
					Prostate Adenocarcinoma	S149	S149Pfs*21	The TP53 S149Pfs*21 mutation is likely oncogenic
					Pancreatic Adenocarcinoma	S149	S149Ffs*32	The TP53 S149Ffs*32 mutation is likely oncogenic
*TTN*	30	TITIN	Q8WZ42	T671, T826, S1571, T3501, S4651, T4659, S7613, S10385, S10781, T12007, T14674, S28157, S28450, S33976	Uterine Endometrioid Carcinoma	T671	*T671S*	Unknown
					Uterine Endometrioid Carcinoma	S1571	*S1571T*	Unknown
					Cutaneous Melanoma	S1571	*S1571F*	Unknown
*MUC16*	19.3	MUC16	Q8WXI7	S12117, S13054, T13833	Uterine Endometrioid Carcinoma	S12117	*S12117N*	Unknown
					Renal Clear Cell Carcinoma	S13054	*S13054T*	Unknown
					Renal Clear Cell Carcinoma	S13054	*S13054F*	Unknown
*PIK3CA*	14.1	PK3CA	P42336	no data				
*CSMD3*	13.3	CSMD3	Q7Z407	no data				
*RYR2*	13.2	RYR2	Q92736	T1468		No overlapping sites		
*LRP1B*	12.7	LRP1B	Q9NZR2	no data				
*SYNE1*	12.2	SYNE1	Q8NF91	S1286, T1951, S3382, T4836, T7270	Serous Ovarian Cancer	T1951	*T1951A*	Unknown
					Cutaneous Melanoma	S3382	*S3382N*	Unknown
*FLG*	11.4	FILA	P20930	na				
*USH2A*	11.2	USH2A	O75445	na				

no data: could not find human protein in *O*-GlcNAc database, na: no *O*-GlcNAc site reported

**Table 2:** *O*-GlcNAc expression and roles in various cancers

Cancer type	Research object	*O*-GlcNAc	OGT	OGA	Major findings	Clinical	References
Cervical cancer	HPV-related cervical tumors and human cervical cancer cell lines	Elevated	Elevated	no significant change	Elevated OGT activated the transcription of HPV E6/E7 and thus enhancing the oncogenic activity of HPV		Zeng et al. (2016)
	The human cervical cancer cell lines	Elevated	Elevated	Na	Elevated OGT not only increased the expression of E6/E7 oncoproteins but also promoted HCF-1-mediated transcriptional activity of the E6/E7 promoter		Xu et al. (2021)
	The human cervical cell lines	Elevated	Elevated	Na	*O*-GlcNAcylation of NF-κB in cervical cancer promoted lung metastasis of cervical cancer by activating CXCR4		Ali et al. (2017)
Breast cancer	Primary breast malignant tumors	Elevated	Elevated	Na	Reduction of *O*-GlcNAcylation inhibited the anchorage-independent growth of breast cancer cells		Champattanachai et al. (2013)
	Breast cancer cell lines	Elevated	Elevated	Na	*O*-GlcNAcylation enhanced the migration/invasion of breast cancer cells in vitro and lung metastasis in vivo		Gu et al. (2010)
	Breast cancer cell lines	Elevated	Elevated	Na	Elevated *O*-GlcNAcylation and OGT levels contributed to cancer cell growth and invasion,		Caldwell et al. (2010)
	Breast cancer cell lines	Elevated	Elevated	Na	Nutrient sensing pathway HBP connected with the SIRT1 deacetylase via *O*-GlcNAcylation to regulate cellular invasion via regulation of FOXM1		Ferrer et al. (2017)
	Breast cancer stem cells	Elevated	Elevated	Na	OGT played a key role in the regulation of breast CSCs in vitro and tumor initiation in vivo		Akella et al. (2020)
	HR + /HER2- luminal breast cancer patient samples	Elevated	Na	Na	Hyper-*O*-GlcNAcylation was associated with poor 10-year DFS in patients with breast cancer	Poor survival	Kuo et al. (2021)
Endometrial cancer	Endometrial cancer patient samples	Na	Elevated	Elevated	The *OGT* and *OGA* expression were significantly higher in tumors of a higher histological grade and associated with the depth of tumor invasion into the myometrium		Krzeslak et al. (2012)
	Endometrial cancer cell lines	Elevated	Na	Na	Hyper-*O*-GlcNAcylation promoted EMT in endometrial cancer cells		Jaskiewicz and Townson (2019)
OV	The human ovarian carcinoma cell lines	Elevated	Elevated	Na	*O*-GlcNAcylation decreased E-cadherin level, thereby inhibiting E-cadherin/catenin complex formation and reducing cell–cell adhesion, leading to cancer cell metastasis		Jin et al. (2013)
	The human ovarian carcinoma cell lines	Elevated	Elevated	Na	*O*-GlcNAcylation augments the motility of ovarian cancer cells via the RhoA/ROCK/MLC signaling pathway		Niu et al. (2017)
Liver cancer	HCC patient samples and cell lines	Elevated	Elevated	Na	*O*-GlcNAcylation of AGER increased its activity and stability to promote the development of HCC under high glucose conditions		Qiao et al. (2016a)
	HCC patient samples and cell lines	Elevated	Elevated	Na	*O*-GlcNAcylation of HDAC1 was overexpressed in HCC, and the progression of HCC can be inhibited by inhibiting the *O*-GlcNAcylation of HDAC1		Zhu et al. (2016)
	Livers of diabetic mice	Elevated	Na	Na	There is positive auto-regulatory feedback between *O*-GlcNAcylation and TRIB2, which might be critical for diabetes-associated liver cancer		Yao et al. (2016)
	HCC patient samples and cell lines	Elevated	Elevated	Na	ACSL4 promoted HCC growth and survival by enhancing *O*-GlcNAcylation and activating mTOR signaling. Conversely, *O*-GlcNAcylation facilitated HCC growth via increasing ACSL4 expression and activating mTOR signaling		Wang et al. (2020)
	NAFLD-HCC patient samples, and liver cancer cell lines	Na	Elevated	Na	OGT played an oncogenic role in NAFLD-associated HCC through regulating palmitic acid and inducing ER stress, consequently activating oncogenic JNK/c-Jun/AP-1 and NF-κB cascades		Xu et al. (2017)
	Samples from patients with HCC recurrence after liver transplantation	Elevated	Elevated	Decreased	*O*-GlcNAcylation was significantly enhanced in the tumor tissues of patients who had suffered from HCC recurrence after LT compared with those who had not. Importantly, low expression of OGA was an independent prognostic factor for predicting tumor recurrence of HCC following LT, especially in patients with low AFP expression	Poor Survival	Zhu et al. (2012)
	HCC patient samples	Elevated	Na	Na	Increased *O*-GlcNAcylation of RACK1 is positively correlated with tumor growth, metastasis, and recurrence in patients with HCC		Duan et al. (2018)
	Liver cancer patient samples and cell lines	Elevated	Na	Na	YAP was *O*-GlcNAcylated at Thr241 thereby antagonizing Hippo pathway-mediated phosphorylation of YAP, thus allowing YAP to promote liver tumorigenesis under diabetes-prone, high-glucose conditions		Zhang et al. (2017b)
CRC	CRC patient samples and the human colon tumor cell lines	Elevated	Elevated	no significant change	*O*-GlcNAcylation enhanced the anchorage-independent growth of colon cancer cells		Mi et al. (2011)
	CRC patient samples	Elevated	Elevated	no significant change	Abnormal *O*-GlcNAc-modified proteins, particularly annexin A2, may be novel biomarkers for CRC		Phueaouan et al. (2013)
	CRC patient samples and the CRC cell lines	Elevated	Na	Na	*O*-GlcNAcylation at Thr236 of YY1 enhanced the expression of SLC22A15 and AANAT in cells and increase the protein stability of YY1 itself to exert its oncogenic effect		Zhu et al. (2019)
	Human CRC cell lines	Elevated	Elevated	Na	Hyper-*O*-GlcNAcylation significantly contributed to tumor proliferation and metastasis and indicate a poor prognosis in patients with CRC	Poor survival	Wu et al. (2019)
	The murine colon carcinoma cells	Elevated	Na	Na	*O*-GlcNAcylation deregulated β-catenin and E-cadherin expression and activity in fibroblast cell lines and this might influence EMT and cell motility, which may further influence tumor development and metastasis		Harosh-Davidovich and Khalaila (2018)
	Human colon cancer cells	Elevated	Elevated	Na	*O*-GlcNAcylation of XIAP at Ser406 is essential for its E3 ubiquitin ligase activity toward specifically OGT		Seo et al. (2020)
	CRC patient samples, CRC cell lines	Elevated	Elevated	Na	ITGA5 overexpression accelerates the progression of CRC, which is closely associated with its enhanced *O*-GlcNAcylation		Yu et al. (2019)
PDAC	Human pancreatic cancer cells	Elevated	Elevated	Decreased	Hyper-*O*-GlcNAcylation played an important role in PDAC cells’ survival and constitutive NF-κB activity		Ma et al. (2013)
	PDAC cells	Elevated	Elevated	Elevated	OGA promotes *OGT* transcription through cooperation with the histone acetyltransferase p300 and transcription factor CCAAT/enhancer-binding protein β (C/EBPβ)		Qian et al. (2018)
	The pancreatic cancer cell lines	Elevated	Elevated	Na	Triptolide-induced cell death in pancreatic cancer is mediated by alteration of *O*-GlcNAcylation of Sp1		Banerjee et al. (2013)
GC	Primary GC patient samples	Elevated	Elevated	Na	*O*-GlcNAcylation was associated with the carcinogenesis and progression of GC	Poor survival	Jang and Kim (2016)
	GC patient samples and cell lines	Elevated	Elevated	Na	Hyper-*O*-GlcNAcylation significantly promoted GC cell proliferation by modulating cell cycle-related proteins and ERK 1/2 signaling	Poor survival	Jiang et al. (2016)
ESCC	ESCC patient samples	Elevated	Elevated	Na	Hyper-*O*-GlcNAcation stabilized proteins, leading to changes in cellular signal transduction and resulting in tumorigenesis and metastasis	Poor survival	Qiao et al. (2012)
	ESCSs, ESCC cell lines	Na	Elevated	Na	OGT in exosomes from ECSCs protected ECSCs from CD8 + T cells through up-regulation of PD-1		Yuan et al. (2021)
CHOL	CHOL patient samples	Elevated	Elevated	Decreased	Hyper-*O*-GlcNAcylation in CHOL tissues was associated with poor patient outcomes	Poor Survival	Phoomak et al. (2012)
PC	PC patient samples and cell lines	Elevated	Elevated	Na	OGT and *O*-GlcNAcylation were elevated in PC cells and required for growth, invasion, angiogenesis, and metastasis		Lynch et al. (2012)
	PC biopsy patient samples	Elevated	Na	Na	Hyper-*O*-GlcNAcylation was associated with decreased OS of patients	Poor Survival	Kamigaito et al. (2014)
	PC cell lines	Elevated	Elevated	Na	*O*-GlcNAcylation enhanced the malignancy of PC cells by inhibiting the formation of the E-cadherin/catenin/cytoskeleton complex		Gu et al. (2014)
	PC patient samples	Na	Elevated	Na	Inhibition of OGT in PC cells resulted in slowing of the cell cycle and a reduction in DNA replication via a MYC-dependent pathway		Itkonen et al. (2013)
BC	Urine obtained from BC patients	Na	Elevated	Elevated	Analysis of urinary content of *OGA* and *OGT* mRNA was useful for bladder cancer diagnostics		Rozanski et al. (2012)
	BC patient samples and cell lines	Elevated	Elevated	Na	Hyper-*O*-GlcNAcylation enhanced oncogenic phenotypes and was involved in DNA damage response in BC		Wang et al. (2018)
	BC patient samples and cell lines	Na	Elevated	Decreased	Knockdown of OGT inhibited cell proliferation, migration, invasion, and induce cell cycle arrest, while these effects were reversed when OGA is inhibited		Jin et al. (2020a)
RCC	RCC patient samples and cell lines	Elevated	Elevated	na	Hyper-*O*-GlcNAcylation was correlated with poor prognosis in RCC patients. OGT knockdown significantly suppressed RCC cell proliferation in vitro and in vivo	Poor survival	Wang et al. (2019)
Lung cancer	Lung cancer patient samples and cell lines	Elevated	Elevated	na	Hyper-*O*-GlcNAcylation increased the growth and invasion of lung cancer cells		Mi et al. (2011)
	LUAD patient samples	Elevated	Elevated	Elevated	High expression of OGT could independently predict poor survival outcomes in patients with stage I LUAD	Poor survival	Lin et al. (2018)
	Lung cancer patient samples and LUAD cell lines	Elevated	Elevated	na	*O*-GlcNAcylation promoted migration and invasion by activating IL-6/STAT3 signaling in lung cancer		Ge et al. (2021a)
SCLC	SCLC patient samples	Elevated	Elevated	Elevated	High OGT and OGA levels were associated with poor prognosis and could be considered new biomarkers of the invasive phenotype of tumor cells	Poor survival	Starska et al. (2015)
CLL	Blood from CLL patients, CLL cells	Elevated	Elevated	Na	Indolent and aggressive clinical behavior of CLL cells were correlated with higher and lower *O*-GlcNAcylation levels, respectively		Shi et al. (2010)
AML	AML patient samples and cell lines	Na	Elevated	Na	Elevated OGT expression was significantly associated with poor OS in patients with AML. Inhibition of OGT inhibited AML cell proliferation and promoted AML cell apoptosis	Poor survival	He et al. (2021)
	AML patient samples and cell lines	Elevated	Elevated	Na	Inhibition of HBP or OGT led to AML cell differentiation and apoptosis		Asthana et al. (2018)
ALL	Pre-B ALL patient samples and cell lines	Elevated	Elevated	Decreased	*O*-GlcNAcylation aggravated pre-B-ALL through regulation of glycolysis via the PI3K/Akt/c-Myc pathway		Zhang et al. (2017a)
DLBC	DLBC patient samples and cell lines	Elevated	Elevated	Na	Elevated OGT levels were associated with poor survival of patients with DLBC. Targeting OGT in DLBC cells inhibited activation of *O*-GlcNAcylation and NF-κB	Poor survival	Pham et al. (2016)
TC	TC patient samples	Decreased	na	Elevated	OGA activity increased in TC in comparison to non-neoplastic lesions and adenomas		Krzeslak et al. (2010)
	Papillary thyroid cancer patient samples and cell lines	Elevated	Elevated	Na	*O*-GlcNAcylation of YAP at Ser109 dramatically inhibited its Ser127 phosphorylation, subsequently promoting tumor aggressiveness	Poor survival	Li et al. (2021a)
GBM	GBM patient samples	Elevated	Elevated	Na	OGT regulates acetate-dependent acetyl-CoA and lipid production in GBM cells by regulating phosphorylation of ACSS2 by CDK5		Ciraku et al. (2022)

**Table 3:** The available inhibitors targeting OGT

Categories	Compound	IC_50_ (μM)	Advantages	Disadvantages	References
Substrate and product analogs	Alloxan	18 ± 1	Cell-permeable	Potential off-target effects and general cellular toxicity	Konrad et al. (2002)
	UDP- S -GlcNAc	93 ± 15	Sub-millimolar inhibitors	Lack of cell permeability	UniProt: a worldwide hub of protein knowledge (2019)
	UDP- C-GlcNAc	41 ± 7	Sub-millimolar inhibitors	Lack of cell permeability, a weak hOGT inhibitor	Dorfmueller et al. (2011)
	C-UDP	9.0 ± 1.0	Sub-millimolar inhibitors	Lack of cell permeability	Dorfmueller et al. (2011)
	UDP-5SGlcNAc	5	Cell-permeable	Affect N-glycosylation in cells and glycan synthesis outside the cells	Gloster et al. (2011)
HTS-derived inhibitors	ST045849	53 ± 7	Highly selective and cell-permeable	Potential off-target effects and cellular toxicity	Kamigaito et al. (2014)
	OSMI-1	2.7	Cell-permeable, not alter cell surface N- or O-linked glycans, on-target activity		Ortiz-Meoz et al. (2015)
Bisubstrate inhibitor	goblin1	18	Can synergize with goblin2 to enhance inhibition	Lack of cell permeability	Borodkin et al. (2014)
	goblin2	40	Can synergize with goblin1 to enhance inhibition	Lack of cell permeability	Borodkin et al. (2014)

Another key hallmark of cancer is the reprogramming of energy metabolism in cancer cells (Hanahan and Weinberg 2011). The Warburg effect (Vander et al. 2009; Liberti and Locasale 2016) allows cancer cells to greatly increase glucose uptake and HBP flux. Therefore, as a downstream pathway of HBP, *O*-GlcNAcylation in cancer cells is largely affected by this particular mode of metabolism. Hyper-*O*-GlcNAcylation has been observed in almost all types of cancer (Ma and Vosseller 2014), including ovarian cancer (Queiroz et al. 2016), cervical cancer (Zeng et al. 2016), breast cancer (Caldwell et al. 2010), endometrial cancer (Krzeslak et al. 2012), liver cancer (Xu et al. 2017), colorectal cancer (Mi et al. 2011), cholangiocarcinoma (Phoomak et al. 2012), pancreatic cancer (Ma et al. 2013), gastric cancer (Jiang et al. 2016), esophageal squamous cell carcinoma (Qiao et al. 2012), bladder cancer, (Jin et al. 2020a) prostate cancer (Lynch et al. 2012), renal cell carcinoma (Wang et al. 2019), lung cancer (Lin et al. 2018), laryngeal cancer (Starska et al. 2015), thyroid papilloma (Li et al. 2021a), chronic and acute lymphoblastic leukemia (Shi et al. 2010; Zhang et al. 2017a) and glioblastoma (Ciraku et al. 2022). In addition to participating in metabolic reprogramming of cancer cells, *O*-GlcNAcylation also linked to various hallmarks of cancer, including cancer cell survival, proliferation, angiogenesis, invasion, metastasis, and epigenetics (Ma and Vosseller 2014). This part focuses on the role of *O*-GlcNAc in cancers from multiple systems (Table 2).

### Cancers in the reproductive system

#### Cervical cancer

Determining the relationship between *O*-GlcNAc and cervical cancer, especially in human papilloma virus (HPV)-related cervical tumors, has attracted increased attention from researchers. Zeng et al. (Zeng et al. 2016) reported a significant upregulation of *O*-GlcNAcylation with increased OGT levels in HPV-induced cervical tumors, while OGA levels were not altered. Subsequently, some studies found that OGT could mediate *O*-GlcNAc modification of host cell factor C1 (HCF-1) to activate HPV E6/E7 transcription, thereby leading to immortalization and transformation of cells (Kim et al. 2016; Scheffner et al. 1990; Roman and Munger 2013). Furthermore, in addition to the observation that *O*-GlcNAcylation promotes tumorigenesis and metastasis by enhancing the oncogenic activity of HPV in vitro and in mouse models, *O*-GlcNAcylation was found to promote lung metastasis of cervical cancer by modifying nuclear factor kappa B (NF-κB) and thus activating C-X-C chemokine receptor 4 (CXCR4) (Ali et al. 2017).

#### Breast cancer

Many studies have focused on relationship between *O*-GlcNAcylation and the development and progression of breast cancer. Gu et al. (Gu et al. 2010) showed that *O*-GlcNAcylation in breast tumors was significantly increased compared with that in adjacent tissue. In addition, *O*-GlcNAcylation was more abundant in metastatic tissues compared with that in original tumor tissues. The authors also revealed that elevated *O*-GlcNAcylation could enhance the migration and invasion of breast cancer cells in vitro and lung metastasis in vivo, possibly by modifying P120 and β-catenin, thus inhibiting the binding of E-cadherin to P120 on the cell surface, thereby reducing intercellular adhesion. Caldwell et al. (Caldwell et al. 2010) showed that elevated *O*-GlcNAcylation and OGT promoted breast cancer cell growth and invasion, in part by regulating the oncogenic transcription factor forkhead box M1 (FOXM1) and multiple FOXM1-specific targets, such as s-phase kinase-associated protein 2 (SKP2) and cyclin-dependent protein kinase inhibitor (P27^Kip1^). Ferrer et al. (Ferrer et al. 2017) revealed that elevated *O*-GlcNAcylation promoted the invasion and metastasis of breast cancer by regulating the sirtuin 1 (SIRT1)/extracellular regulated kinase (ERK)/FOXM1 axis. Notably, Akella et al. (Akella et al. 2020) found that OGT/*O*-GlcNAc is essential and sufficient for maintaining breast cancer stem-like cells phenotype in breast cancer cells in vitro and plays a critical role in tumor-initiating potential in vivo. In addition, Kuo et al. (2021) showed that hyper-*O*-GlcNAcylation is associated with poor 10-year disease-free survival (DFS) in patients with breast cancer. These studies suggest that *O*-GlcNAcylation acts as a stimulating factor in the occurrence, invasion, metastasis, recurrence, and prognosis of breast cancer.

#### Endometrial cancer

Previous studies have determined the relationship between *OGT* and *OGA* mRNA expression levels and the clinical and pathological features of endometrial cancer (Krzeslak et al. 2012). The results showed that higher *OGT* and *OGA* levels were associated with higher histological grades and depth of tumor invasion into the myometrium. Research from Jaskiewicz et al. (Jaskiewicz and Townson 2019) showed that hyper-*O*-GlcNAcylation could promote cell proliferation, induce cytoskeletal reorganization, and induce epithelial-mesenchymal transition (EMT), thereby enhancing cell invasion. Interestingly, hypo-*O*-GlcNAcylation could also enhance the expression of the EMT-related gene *WNT5B* (encoding Wnt family member 5B), but decreased the overall proliferation and migration ability of the cells. These results suggested that breaking the *O*-GlcNAc cycle in endometrial cancer cells promotes EMT at the molecular and cellular levels, but only high *O*-GlcNAcylation causes proliferation, migration, and cytoskeletal reorganization of cells.

#### Ovarian cancer

Jin et al. (Jin et al. 2013) reported higher levels of *O*-GlcNAcylation and *OGT* mRNA in highly metastatic ovarian cancer HO-8910PM cells compared with low metastatic OVCAR3 cells and revealed the underlying molecular mechanism: hyper-*O*-GlcNAcylation decreased E-cadherin expression, thereby inhibiting E-cadherin/catenin complex formation, which reduced cell–cell adhesion, leading to cancer cell metastasis. Niu et al. (Niu et al. 2017) reported increased levels of *O*-GlcNAc and OGT in ovarian carcinoma cell lines and showed that *O*-GlcNAcylation of GTP-bound RhoA and modulator of VRAC current 1 (MLC1) phosphorylation might activate the RhoA/Rho associated coiled-coil containing protein kinase (ROCK)/MLC1 pathway to enhance ovarian cancer cell mobility, thus contributing to ovarian cancer cell migration and invasion.

### Cancers in the digestive system

#### Liver cancer

Numerous studies have noted that *O*-GlcNAcylation is elevated in liver cancer, especially in hepatocellular carcinoma (HCC) (Qiao et al. 2016a; Zhu et al. 2016, 2012). Diabetes mellitus is an important risk factor for the development of liver cancer (Mukherjee et al. 2015); therefore, increasing numbers of studies have focused on the link between *O*-GlcNAcylation and high glucose stimulated liver cancer. Zhang et al. (Zhang et al. 2017b) reported that under high glucose condition, Yes-associated protein (YAP) can be *O*-GlcNAcylated at Thr241, thereby enhancing its expression, stability, and function, leading to the transformed phenotype of HCC cells. Importantly, they demonstrated that *O*-GlcNAcylation of YAP is essential during liver tumorigenesis induced by high glucose. Qiao et al. (Qiao et al. 2016a) found that the advanced glycosylation end product-specific receptor (AEGR) can increase its activity and stability through *O*-GlcNAcylation at Ser73, thus promoting the development of liver tumors under high glucose conditions.

*O*-GlcNAc can also modify many oncoproteins, thereby promoting liver tumorigenesis. Yao et al. (Yao et al. 2016) revealed that *O*-GlcNAcylation of tribbles pseudokinase 2 (TRIB2) enhances protein stability, which in turn promotes HBP and *O*-GlcNAcylation, thus maintaining the transformed phenotype of hepatoma cells. Another study showed that histone deacetylase-1 (HDAC1) is over *O*-GlcNAcylated in HCC and the progression of HCC can be reduced by inhibiting HDAC1 *O*-GlcNAcylation (Zhu et al. 2016). In non-alcoholic fatty liver disease-related hepatocellular carcinoma (NAFLD-HCC), researchers observed elevated OGT levels in patients with NAFLD-HCC and NAFLD-HCC cell lines and revealed that OGT could regulate lipid metabolism, thereby activating endoplasmic reticulum (ER) stress, and the JUN N-terminal kinase (JNK)/Jun proto-oncogene, AP-1 transcription factor subunit (c-Jun)/activator protein 1 (AP-1), and NF-κB cascades during the development of NAFLD-HCC (Xu et al. 2017); indeed, the latter has been shown to be a cancer-promoting factor in HCC (Han and Roman 2006). In addition, orthotopic HCC xenograft models indicated that OGT significantly promotes HCC lung metastasis by inhibiting E-cadherin and enhancing vimentin expression.

Liver transplantation (LT) is currently an effective way to treat patients with early HCC and cirrhosis. However, the frequent recurrence of tumors after LT still represents a great obstacle to the long-term survival of patients (Zheng et al. 2008). Zhu et al. (Zhu et al. 2012) observed significantly higher *O*-GlcNAcylation and lower OGA levels in tumor tissues from patients that suffered from HCC recurrence after LT compared with those who did not. Moreover, they revealed that elevated *O*-GlcNAcylation resulted in decreased E-cadherin and increased matrix metalloproteinase (MMP)1, MMP2, and MMP3 expression, thereby promoting migration and invasion. The study also identified lower OGA expression as an independent predictor for HCC tumor recurrence after LT, especially in patients with low alpha fetoprotein (AFP) expression. *O*-GlcNAcylation affects chemoresistance in HCC. Duan et al. (Duan et al. 2018) confirmed that *O*-GlcNAcylation of ribosome-activated C kinase 1 (RACK1) at Ser122 in HCC cells would lead to acquired chemoresistance and recurrence in patients. Taken together, these studies indicate that *O*-GlcNAcylation affects the occurrence, progression, and recurrence of HCC in a complex manner.

#### Colorectal cancer

Hyper-*O*-GlcNAcylation and elevated OGT levels were observed in colorectal cancer (CRC) tissues compared with those in adjacent normal tissues, while OGA expression showed no significant difference between tumor and normal tissues. (Mi et al. 2011; Phueaouan et al. 2013; Steenackers et al. 2016) Hyper-*O*-GlcNAcylation participates in the progression of CRC through multiple pathways. Recently, Zhu et al. (Zhu et al. 2019) showed that *O*-GlcNAcylation of transcription factor YIN-YANG-1 (YY1) at Thr236 could enhance the expression of solute carrier family 22 member 15 (SLC22A15) and aralkylamine *N*-acetyltransferase (AANAT) and increased its own protein stability, thereby exerting an oncogenic effect in CRC cells. Wu et al. (2019) found that RNA helicase p68/DEAD-box helicase 5 (DDX5) could be *O*-GlcNAcylated to enhance its stability, thus increasing the activation of the protein kinase B (AKT)/mechanistic target of rapamycin kinase (mTOR) signaling pathway, leading to the promotion of the malignant development of CRC. Lefebvre et al. (Olivier-Van et al. 2014, 2012) revealed that *O*-GlcNAcylation of β-catenin is the molecular event that links the glucose metabolism deregulation observed in metabolic disorders and the development of CRC. They observed that human colon tumors and colons from mice fed high-carbohydrate diets exhibited higher amounts of β-catenin and *O*-GlcNAc relative to healthy tissues and mice fed a standard diet, respectively. Subsequently, through analysis of β-catenin *O*-GlcNAcylation mutants, they identified Thr41 as the most crucial residue that controls the β-catenin degradation rate and found that β-catenin was *O*-GlcNAcylated at Thr41 thereby reducing its degradation and thus affecting the development of CRC. Harosh-Davidovich et al. (Harosh-Davidovich and Khalaila 2018) found that hyper-*O*-GlcNAcylation could not only enhance the expression of β-catenin and E-cadherin, but also increased the rate of β-catenin entry into the nucleus and enhanced its transcriptional activity, thereby promoting cell motility and tumorigenicity in CRC. Yu et al. (Yu et al. 2019) reported that *O*-GlcNAcylation of integrin α5 (ITGA5) would enhance its stability, thus promoting CRC cell proliferation and tumorigenesis, and reducing apoptosis. *O*-GlcNAcylation can also affect colon cancer stem cells (CCSCs). Guo et al. (Guo et al. 2017) demonstrated that *O*-GlcNAcylation in CRC could affect the epigenetic inheritance of CCSCs by regulating the transcription factor MYB proto-oncogene like 1 (MYBL1), thus regulating colon carcinogenesis.

#### Pancreatic cancer

In normal cells, OGT and OGA can regulate each other's activity and expression at the transcriptional and post-translational levels to maintain cellular *O*-GlcNAc levels in the "optimal region" (Yang and Qian 2017). However, studies have observed an increase in *O*-GlcNAcylation and OGT, and a decrease in OGA, in human pancreatic ductal adenocarcinoma (PDAC) (Ma et al. 2013). Qian et al. (Qian et al. 2018) revealed that this dysregulation was caused by the disruption of transcriptional regulation homeostasis in pancreatic cancer and proved that it was the abnormal activation of ERK signal transduction in cells that affected OGA-mediated *OGT* transcription, ultimately resulting in increased *OGT* expression (Wang et al. 1999; Liptay et al. 2003). *O*-GlcNAcylation was associated with immune evasion in pancreatic cancer. Shang et al. (Shang et al. 2021) observed an enhanced folate cycle, higher concentration of UDP-GlcNAc, and higher cMYC *O*-GlcNAcylation in patients with pancreatic cancer, which was proven to increase *PDL1* (encoding programmed cell death 1 ligand 1) transcription, leading to immune escape. OGT can be used as a potential treatment target for pancreatic cancer. Triptolide, a diterpene epoxide from the Chinese plant *Tripterygium wilfordii*, targets OGT, resulting in downregulation of heat shock factor 1 (HSF1) and other heat shock proteins (HSPs), ultimately leading to tumor cell death (Banerjee et al. 2013). These studies suggested that dysregulation of *O*-GlcNAcylation is strongly associated with pancreatic cancer and could serve as a potential therapeutic target.

#### Gastric cancer

Previous studies have observed progressively higher OGT and *O*-GlcNAcylation levels as gastric cancer (GC) progresses: *O*-GlcNAcylation and OGT levels were higher in GC with the intestinal type, higher pathological and clinical stage, and more lymph node metastasis (Jang and Kim 2016). *O*-GlcNAcylation promotes GC progression through multiple pathways. Zhang et al. (Zhang and Chen 2016) revealed that *O*-GlcNAcylation enhanced GC cell invasion through the phosphatidylinositol-4,5-bisphosphate 3-kinase (PI3K)/AKT pathway. Cheng et al. (Cheng et al. 2016a) reported that *O*-GlcNAcylation of intercellular scaffold protein guanine nucleotide binding protein (G protein), beta polypeptide 2-like 1 (GNB2L1) could promote its degradation, thereby blocking the inhibitory effect of GNB2L1 on gastric cancer cell migration, and eventually leading to metastases. Jiang et al. (Jiang et al. 2016) found that *O*-GlcNAcylation can regulate the cell cycle and ERK 1/2 pathway, thereby promoting the proliferation of GC cells. These studies further confirmed the adverse effect of hyper-*O*-GlcNAcylation in GC.

#### Esophageal cancer and cholangiocarcinoma

Qiao et al. (Qiao et al. 2012) reported higher *O*-GlcNAc and OGT levels in patients with esophageal squamous cell carcinoma (ESCC) compared with that in normal samples, they also revealed (Qiao et al. 2016b) that hyper-*O*-GlcNAcylation could promote tumorigenesis and metastasis of ESCC by raising the stability and expression of MMP9, and changing cellular signal transduction. Similar to pancreatic cancer, Yuan et al. (Yuan et al. 2021) reported that hyper-*O*-GlcNAcylation could improve the self-renewal capacity of esophageal cancer stem cells (ECSCs) and promote the high expression of programmed cell death 1 (PD1) in CD8 + T cells, resulting in immune escape. Higher *O*-GlcNAcylation and OGT levels, and lower OGA levels, were observed in cholangiocarcinoma compared with those in normal bile ducts, and this change led to poor prognosis of patients with cholangiocarcinoma (Phoomak et al. 2012).

### Cancers in the urinary system

#### Prostate cancer

Lynch et al. (Lynch et al. 2012) first reported higher *O*-GlcNAcylation and OGT levels in prostate cancer (PC) tissues and cell lines compared with those in normal samples; they proved that this is related to the invasive phenotype and ability of PC cells and worse clinical outcomes. Furthermore, they reduced vascular endothelial growth factor (VEGF)-mediated angiogenesis of PC cells by targeting OGT. Gu et al. (Gu et al. 2014) revealed that hyper-*O*-GlcNAcylation promotes PC invasion by inhibiting the formation of the E-cadherin/catenin/cytoskeleton complex, rather than affecting *CDH1* (encoding E-cadherin) mRNA levels.

Another study (Li et al. 2017) revealed that OGT could bind to the BMI1 proto-oncogene, polycomb ring finger (BMI-1)/protein regulator of cytokinesis 1 (PRC1) complex and modify BMI-1 at Ser255, thereby promoting its stability and oncogenic activity, resulting in inhibition of the tumor protein 53 (TP53), phosphatase and tensin homolog (PTEN), and cyclin dependent kinase Inhibitor (CDKN)1A/CDKN2A pathways. The importance of c-MYC for PC has been demonstrated (Hawksworth et al. 2010; Gurel et al. 2008). Slawson et al. (Slawson and Hart 2011) showed that *O*-GlcNAcylation of c-MYC at Thr58 could inhibit phosphorylation at nearby Ser62, thereby stimulating the growth of tumor cells. Itkonen et al. (Itkonen et al. 2013) found that inhibition of OGT in PC cells resulted in slowing of the cell cycle and a reduction in DNA replication via a MYC-dependent pathway, thereby reducing tumor growth. These studies all illustrated the important role played by *O*-GlcNAcylation in PC and the potential of OGT as a target to treat PC.

#### Bladder cancer and renal cell carcinoma

A previous study reported that the analysis of OGT and OGA content in urine might contribute to the diagnosis and grading of bladder cancer (BC) (Rozanski et al. 2012). OGT was found in most urine samples with BC, while OGT was not detected in the urine of healthy individuals. In addition, higher OGT and lower OGA levels were observed in the urine from patients with a higher tumor grade, although there were no differences in OGA levels in urine between healthy individuals and patients. Wang et al. (Wang et al. 2018) observed hyper-*O*-GlcNAcylation and increased OGT levels in BC tissues and cell lines compared with those in normal samples. In addition, they revealed that inhibiting OGT reduced BC cell proliferation and growth, triggered apoptosis, and led to cell cycle arrest, probably via increased autophagy. This was confirmed by a subsequent study (Jin et al. 2020b): Blockade of *O*-GlcNAcylation induced autophagy in BC cells through an mTOR-independent pathway. Jin et al. (Jin et al. 2020a) revealed that hyper-*O*-GlcNAcylation can promote the malignant phenotype of BC cells, while knockdown of *OGT* could reverse these effects. Recently, Chen et al. (Chen et al. 2021) reported similar results: Inhibiting OGT led to downregulation of the cell cycle-related protein nucleolar and spindle associated protein 1 (NUSAP1), thereby inhibiting the malignant progression of BC. Melatonin has previously been shown to inhibit the growth of BC (Reiter et al. 2017). Wu et al. (2021) further revealed the anti-prostate cancer mechanism of melatonin: Melatonin inhibits *O*-GlcNAcylation of cyclin-dependent kinase 5 (CDK5) at Thr246, thus promoting its degradation, and inhibiting the tumor-promoting effect of CDK5 on BC cells.

Wang et al. (Wang et al. 2019) first observed significantly higher OGT and *O*-GlcNAcylation levels in renal cell carcinoma (RCC) cell lines and tissues compared with those in normal samples, and found that hyper-*O*-GlcNAcylation was associated with higher grade and poor prognosis in patients. In addition, they revealed that knockdown of *OGT* in RCC cells could downregulate the epidermal growth factor receptor (EGFR) and PI3K/AKT pathways, thereby inhibiting the migration, invasion, and vascularization of RCC cells. Together, these studies demonstrated the potential of *O*-GlcNAcylation as a predictor and treatment target for BC and RCC.

### Cancers in the respiratory system

#### Lung cancer and laryngeal cancer

Many studies have reported the role of *O*-GlcNAcylation in malignant tumors of the respiratory system. Mi et al. (Mi et al. 2011) not only observed elevated OGT and *O*-GlcNAcylation in lung cancer, but also indicated that hyper-*O*-GlcNAcylation would increase the growth and invasion of lung cancer cells. Lin et al. (Lin et al. 2018) showed a clinical relationship between OGT and lung cancer: Hyper-*O*-GlcNAcylation independently predicted a worse prognosis in patients with stage I lung adenocarcinoma. An inflammatory microenvironment is highly correlated with tumor initiation and malignant progression (Hanahan and Weinberg 2011; Pietila et al. 2016). Ge et al. (Ge et al. 2021a) confirmed that the inflammatory factor interleukin 6(IL-6), released by inflammatory cells or tumor cells, increased OGT expression in lung cancer cells though the NF-κB p65 signaling pathway, leading to migration and invasion.

Squamous cell laryngeal cancer (SCLC) is another respiratory malignancy. Researchers have observed hyper-*O*-GlcNAcylation and elevated OGT and OGA in SCLC samples compared with those in the normal laryngeal mucosa. In addition, this change is closely related to worse clinical outcomes of patients, including larger tumor size, higher pathological grade, more lymph node metastasis, and a higher recurrence rate.

### Cancers in the hematopoietic system

#### Chronic lymphocytic leukemia

In addition to solid tumors, there are many reports about the role of *O*-GlcNAcylation in various blood cancers (Spaner 2021). Shi et al. (Shi et al. 2010) reported increased *O*-GlcNAcylation and OGT in chronic lymphocytic leukemia (CLL) cells compared with those in normal lymphocytes, and also revealed that this is caused by higher levels of the sugar donor UDP-GlcNAc. Notably, they found that hyper-*O*-GlcNAcylation appeared to be closely related to an indolent clinical course: Elevated *O*-GlcNAcylation can inhibit JNK signaling, weaken the response of CLL cells to proliferation signals, and hinder the division of CLL cells. Interestingly, the results of their study are somewhat contradictory to those in other cancers, because hyper-*O*-GlcNAcylation usually means more aggressive clinical behavior in solid tumors. However, similar to CLL, hyper-*O*-GlcNAcylation was also observed in chemosensitive ovarian cancer tissues compared with that in chemoresistant ovarian cancer tissues (Zhou et al. 2018). Another study explained the link between *O*-GlcNAcylation and the indolent clinical behavior of CLL (Lode et al. 2016; Yang et al. 2006): *O*-GlcNAcylation of p53 at Ser149 can inhibit cancer progression by stabilizing wild-type p53 and activating the intact p53 pathway. From a therapeutic aspect, Tomic et al. (Tomic et al. 2013) found that the anti-CLL activity of resveratrol (RSV) was associated with the reduction of *O*-GlcNAcylated proteins: RSV rapidly downregulated *O*-GlcNAcylation levels in CLL cells; the authors speculated that this might be a result of proteasomal activation.

#### Other hematological malignancies

**Fig. 6:**
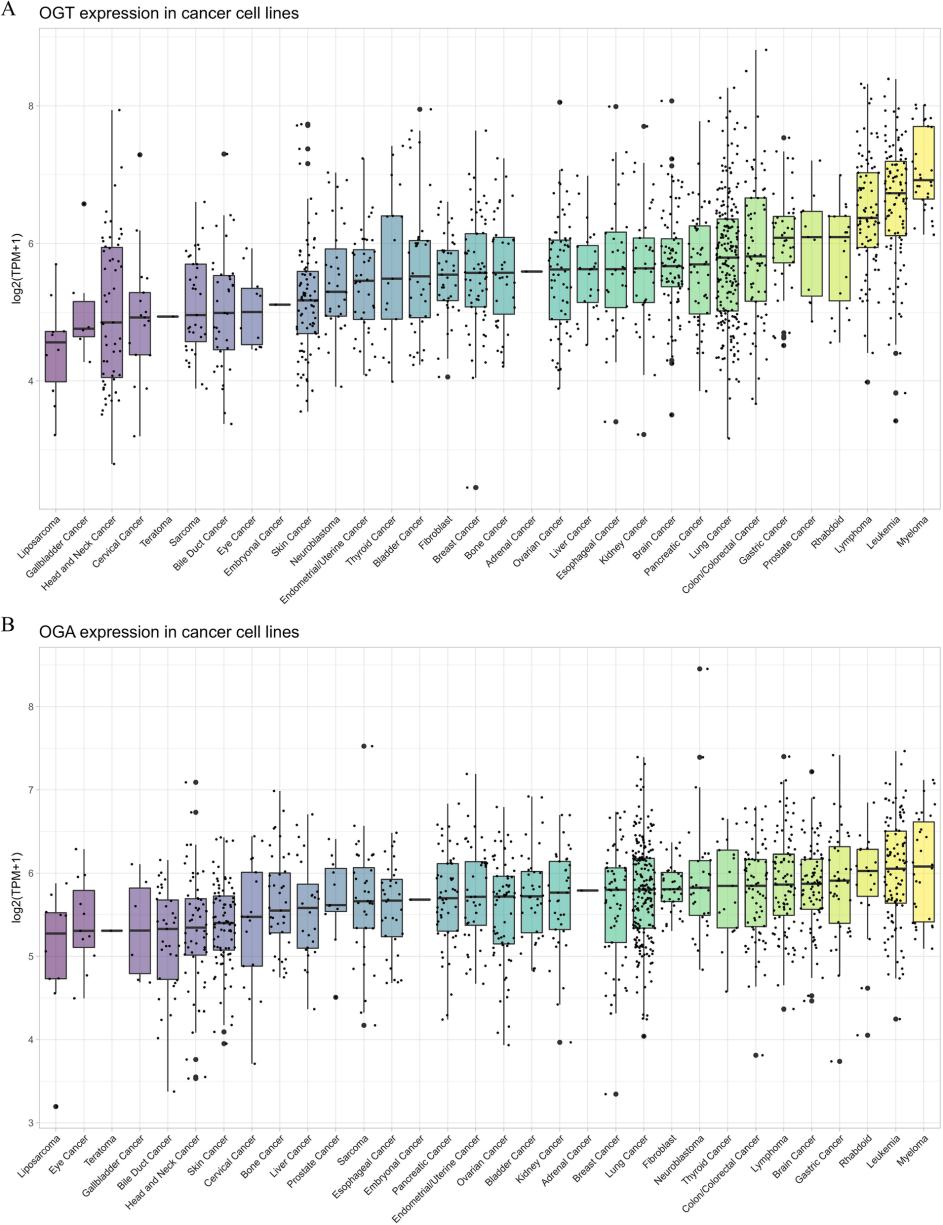
*O*-GlcNAc modifying enzymes expression in different cancer cell lines. Compared with other solid tumors, cancer cell lines of hematological malignancies showed higher expression levels of OGT and OGA. (**A**) *OGT* expression in different cancer cell lines. **B** *OGA* expression in different cancer cell lines. Data were downloaded from the DepMap-portal (https://depmap.org/portal). Each dot represents a cell line and the black bar graphs are median of expression in that tissue type

Besides CLL, abnormal *O*-GlcNAcylation has also been reported in myelodysplastic syndrome (MDS) (Li et al. 2021b), acute myeloid leukemia (AML) (He et al. 2021), acute lymphoblastic leukemia (ALL) (Zhang et al. 2017a), and diffuse large B-cell lymphoma (DLBC) (Pham et al. 2016). Interestingly, higher OGT and OGA expression levels were observed in tumor samples and cell lines from hematological malignancies compared with those in other solid tumors (Itkonen et al. 2021). In various cancer cell lines, the expression levels of OGT and OGA rank first and second in myeloma and leukemia, respectively (Fig. 6); in tumor samples from the TCGA database, the expression of OGT and OGA in LAML was much higher than that in other cancers (Fig. 3). Some studies have revealed the role of *O*-GlcNAcylation in blood cancers. Asthana et al. (Asthana et al. 2018) observed higher OGT and GFAT levels in primary AML cells and revealed that inhibiting the activity of HBP using the GFAT inhibitor 6-diazo-5-oxo-l-norleucine (DON), or using the OGT inhibitors OSMI-1 (OGT with a small molecule inhibitor) and BADGP (benzyl-2-acetamido-2-deoxy-α-d-galactopyranoside), can induce AML cell differentiation and apoptosis, but do not affect normal cells. Chemoresistance in AML is also associated with elevated *O*-GlcNAcylation. Liu et al. (Liu et al. 2018) reported that hyper-*O*-GlcNAcylation induced by chemotherapy drugs, such as doxorubicin (DOX) and camptothecin (CPT), can cause chemoresistance, thereby weakening the therapeutic effect, while targeting OGT enhanced the effect of chemotherapy. Zhang et al. (Zhang et al. 2017a) observed higher OGT and *O*-GlcNAcylation levels, and lower OGA levels, in pre-B acute lymphoblastic leukemia (pre-B-ALL) samples compared with those in healthy donors. Moreover, they demonstrated that hyper-*O*-GlcNAcylation aggravated pre-B-ALL by modulating glycolysis via the PI3K/AKT/c-MYC pathway. Interestingly, many previous studies have shown that elevated OGT is associated with tumor progression. However, Inoue et al. (Inoue et al. 2018) revealed the predictive and inhibitory effect of OGT in hematopoietic malignancies. They showed that additional sex combs like transcriptional regulator 1 (ASXL1) could be stabilized by *O*-GlcNAcylation at Ser199, allowing it to act as a hematopoietic malignant tumor suppressor. Furthermore, they showed that OGT has tumor suppressive activity in myeloid malignancies, especially in the presence of *ASXL1* mutations (Abdel-Wahab et al. 2011; Bejar et al. 2011; Gelsi-Boyer et al. 2009; Yoshida et al. 2011). These results suggested that *O*-GlcNAcylation regulates hematopoietic malignancies in a complex way, and more studies are needed to clarify its role.

### Cancers in the endocrine system

#### Thyroid cancers

Some studies have reported the role of *O*-GlcNAcylation in thyroid cancer (TC). Krześlak et al. (Krzeslak et al. 2010) observed the elevated OGA and decreased *O*-GlcNAcylation levels in TC samples compared with those in non-neoplastic lesions and adenocarcinoma. Yu et al. (Cheng et al. 2016b) revealed that hyper-*O*-GlcNAcylation improves TC cell viability and enhances its growth, migration, and invasion, thereby enhancing the malignant phenotype of TC. Krześlak et al. (Krzeslak et al. 2011) showed that elevated *O*-GlcNAcylation in thyroid anaplastic cancer cells could promote their proliferation through the insulin-like growth factor (IGF-1)-AKT-GSK3β-cyclin D1 pathway. Zhang et al. (Zhang et al. 2015) found that elevated *O*-GlcNAcylation could enhance the invasion of TC cells, in part through PI3K/AKT signaling. Recently, Li et al. (Li et al. 2021a) revealed that YAP, a core component of the Hippo pathway, was *O*-GlcNAcylated at Ser109, thereby inhibiting phosphorylation at Ser127. This allowed YAP to act as an oncogenic transcriptional coactivator to promote TC growth and metastasis, thereby influencing the recurrence-free survival and clinicopathological characteristics of patients with TC (Liu et al. 2017, 2019b). These studies suggested that high levels of *O*-GlcNAcylation are also characteristic of malignant TC, and targeting *O*-GlcNAcylation might be a potential strategy to treat TC.

### Cancers in the nervous system

#### Brain cancer

Recently, the role of *O*-GlcNAcylation in brain tumors has attracted the attention of researchers. Lorela Ciraku et al. (Ciraku et al. 2022) reported that OGT and *O*-GlcNAcylation levels are elevated in glioblastoma (GBM) tissues and cells. In addition, they revealed that elevated *O*-GlcNAcylation in GBM cells regulates acetate-dependent acetyl-CoA and lipid production through the OGT/CDK5/acetyl-CoA synthetase 2 (ACSS2) pathway to adapt to lack of lipid availability in the brain environment. Specifically, elevated OGT in GBM cells increases the phosphorylation of ACSS2 at Ser267 in a CDK5-dependent manner, thereby reducing polyubiquitination and degradation to increase its stability. Chen et al. (Chen et al. 2022) observed the elevated *O*-GlcNAc levels in human samples of Sonic hedgehog (Shh)-subtype medulloblastoma and revealed that OGT in granule neuron precursors (GNPs) may contribute to medulloblastoma oncogenesis by activating the Shh signaling pathway via *O*-GlcNAcylation at S355 of GLI family zinc finger 2 (Gli2). These studies reveal the important role of *O*-GlcNAc signaling in brain cancer and deserve further exploration.

## *O*-GlcNAc and cancer therapy

*O*-GlcNAcylation, as an emerging but ubiquitous protein PTM, has attracted great interest from researchers since it was reported in the early 1980s. To date, numerous publications have addressed its important functions, regulatory mechanisms, and links with human diseases. Although we lack detailed knowledge of the mechanisms, hyper-*O*-GlcNAcylation is a common feature in various cancers, and thus represents a promising potential target for cancer diagnosis and treatment.

### *O*-GlcNAc as a biomarker for cancer diagnosis and prognosis

Many malignant tumors are characterized by difficult early diagnosis, poor prognosis, and easy recurrence, which poses a great challenge for their clinical treatment (Hanahan and Weinberg 2011). Therefore, identifying a reliable biomarker that can be used for early diagnosis, prognosis, and confirmation of recurrence has become an important research direction in cancer treatment. OGT and OGA expression levels have been assessed in patients and normal samples of major human cancers (Cancer Cell Line Encyclopedia Consortium 2015; UniProt Consortium 2019) (Fig. 3). The results showed that OGT and OGA expression levels changed in almost all cancers to varying degrees, suggesting that *O*-GlcNAcylation level and the levels of *O*-GlcNAc circulating enzymes might have significance for early cancer screening and prognosis. In fact, many studies have reported that hyper-*O*-GlcNAcylation often indicates poor prognosis of patients, shorter disease-free survival, and tumor recurrence: Kuo et al. (2021) found that high levels of *O*-GlcNAcylation was an important independent predictor of poor 10-year DFS for HR lysine demethylase and nuclear receptor corepressor (HR)+/human epidermal growth factor receptor 2 (HER2)-luminal breast cancer, and the predictive effect and potential can be greatly enhanced when combined with pyruvate kinase isoenzyme M2 (PKM2). Rozanski et al. (Rozanski et al. 2012) indicated that detecting *OGA* and *OGT* mRNA levels in urine might be helpful in the diagnosis of BC. Kamigaito et al. (Kamigaito et al. 2014) showed that OGT overexpression occurred in 39% of prostate cancer specimens from 56 patients who did not receive hormone therapy, and this OGT overexpression correlated significantly with reduced OS. Furthermore, Zhu et al. (Zhu et al. 2012) revealed that elevated *O*-GlcNAcylation and decreased OGA expression might indicate the recurrence of HCC after LT, and this predictive effect is more pronounced in patients with low AFP levels. Other studies indicated that the level of *O*-GlcNAcylation or OGT can be used as a biomarker to predict poor prognosis of LUAD, laryngeal carcinoma, CHOL, ESCC, GC, CRC, RCC, AML, DLBC, and TC (Table 2). These studies fully illustrate the great potential of *O*-GlcNAc as biomarkers for early diagnosis, prognosis, and confirmation of recurrence of cancer.

### *O*-GlcNAc as target for cancer therapeutics

Given the adverse roles of dysregulated *O*-GlcNAcylation, especially hyper-*O*-GlcNAc, in cancers, such as promoting the growth, proliferation, invasion, and metastasis of cancer cells, inducing cancer angiogenesis, promoting the malignant phenotype of cancer, and promoting chemoresistance (Ma and Vosseller 2014), targeting *O*-GlcNAcylation is an attractive approach for clinical anticancer therapies.

Currently developed chemical drugs, therapeutic antibodies, or aptamers mainly regulate the *O*-GlcNAc pathway through two strategies to achieve therapeutic effects. The first strategy is to target the enzymes OGT and OGA directly for cancer therapy. The change in intracellular *O*-GlcNAcylation levels depends largely on change of these two enzymes, which leads to dysregulation of *O*-GlcNAcylation and the occurrence and progression of diseases. Thus, the adverse effects caused by dysregulated *O*-GlcNAcylation can be largely repaired or reversed by regulating the levels of OGT or OGA. Over the past few decades, investigators have developed various inhibitors against OGT, which belong to three categories: Substrate and product analogs (Dorfmueller et al. 2011), high-throughput screening (HTS)-derived inhibitors (Gross et al. 2005), and bisubstrate inhibitors (Borodkin et al. 2014) (Table 3). Alloxan was the first reported inhibitor of human OGT (Konrad et al. 2002). It is a uracil analog that can inhibit OGT activity, thus blocking hyper-*O*-GlcNAcylation in cells in a dose-dependent manner, presumably by binding to the uracil binding pocket or by covalent modification of cysteine residues (Konrad et al. 2002). However, reactive oxygen species (ROS) and free radicals produced by Alloxan during its involvement in extracellular redox processes can cause a large number of off-target effects and damage cellular structures, such as mitochondria and lysosomes (Zhang et al. 1992). Researchers have reported that UDP-S-GlcNAc, UDP-C-GlcNAc, and C-UDP act as OGT inhibitors (Dorfmueller et al. 2011). Notably, they are sub-millimolar inhibitors but are not cell-permeable. Gloster et al. (Gloster et al. 2011) reported that 5SGlcNAc and its analog Ac-5SGlcNAc could be recovered by cells and processed by the HBP to generate an efficient OGT inhibitor, UDP-5SGlcNAc. Importantly, some experiments have confirmed its anti-cancer roles in breast and prostate cancer, including inhibiting the proliferation, invasion, and angiogenesis of cancer cells. In addition, it can also promote metabolic stress and apoptosis by regulating the oncogenic proteins FOXM1, c-MYC, and hypoxia inducible factor 1 subunit alpha (HIF-1α) (Caldwell et al. 2010; Lynch et al. 2012; Itkonen et al. 2013; Ferrer et al. 2014; Sodi et al. 2015). However, Ac-5SGlcNAc affects the intracellular UDP-GlcNAc repertoire by hijacking the HBP pathway, thereby affecting *N*-glycosylation in cells and glycan synthesis outside the cell (Ortiz-Meoz et al. 2015).

The generation of another class of OGT inhibitors depends on high-throughput screening of large drug compound libraries. Using this approach, many OGT inhibitors have been developed (Gross et al. 2005), including the commercially available small molecule inhibitor ST045849. Itkonen et al. (Itkonen et al. 2016) confirmed the anti-cancer roles of ST045849: They found that inhibition of intracellular OGT activity in prostate cancer cells by ST045849 resulted in complete depletion of intracellular alanine, thereby inhibiting cancer cell viability and growth rate, and continuously induced the death of prostate cancer cells. OSMI-1 is another HTS-derived cell-permeable OGT inhibitor that inhibits *O*-GlcNAcylation, but does not alter the glycan structure on the cell surface (Ortiz-Meoz et al. 2015). OSMI-1 plays an anti-cancer role in various cancers: Lee et al. (2020) revealed that combined treatment using OSMI-1 and tumor necrosis factor (TNF)-related apoptosis inducing ligand (TRAIL) could synergistically enhance TRAIL-induced apoptosis through caspase-8 activation. In addition, OSMI-1 could induce apoptosis by blocking NF-κB signaling and activating the ER stress response, which enhanced the sensitivity of human colon cancer cells to TRAIL-induced cell death. OSMI-1 can also enhance the therapeutic effect of chemotherapeutic drugs. Lee et al. (2020) indicated the combination therapy of OSMI-1 and DOX significantly enhanced apoptosis and DOX-induced cell death of HCC cells by synergistically activating TP53 and mitochondrial B-cell CLL/lymphoma 2 (BCL2) pathways. Makwana et al. (Makwana et al. 2020) and Liu et al. (Liu et al. 2018) showed similar results in prostate cancer and breast cancer. In fact, more HTS-derived OGT inhibitors exist. Martin et al. (Martin et al. 2018) described the structure-based evolution of small molecule inhibitors of OGT and reported three cell-permeable compounds, later termed OSMI-2, OSMI-3, and OSMI-4. Among them, OSMI-2 acts as a rapid-acting OGT inhibitor in combination with anti-androgens to target MYC-dependent prostate cancer cells (Itkonen et al. 2019). Bisubstrate inhibitors against OGT mainly refer to two novel compounds, goblin1 and goblin2, which achieve selective inhibition by replacing the GlcNAc moiety of UDP-GlcNAc with a receptor peptide (Borodkin et al. 2014). However, there are still many limitations to the clinical application of such compounds, mainly because of their lack of cell permeability. Therefore, there is an urgent need for an OGT inhibitor with better specificity, potency, and cell permeability, both for laboratory studies and clinical cancer therapy.

The second strategy of using *O*-GlcNAcylation as a cancer therapeutic target is more targeted: changing the *O*-GlcNAc moieties on specific target proteins to achieve the therapeutic effect. This is quite attractive, because it can avoid the "accidental injury" caused by the alteration of global *O*-GlcNAcylation levels by targeting *O*-GlcNAc cycle enzymes. *O*-GlcNAcylation, as a widespread intracellular PTM, is involved in many aspects of the regulation of cellular life activities, and changes in its circulating enzymes could affect *O*-GlcNAcylation on thousands of proteins, many of which are not disease-related. Arbitrarily changing the *O*-GlcNAcylation of these proteins might have serious consequences. So far, a number of new technologies have emerged that can support the implementation of this idea, such as gene editing techniques, which eliminate the corresponding *O*-GlcNAcylation sites by introducing point mutations in proteins of interest; many laboratories have taken this approach when studying *O*-GlcNAcylation (Duan et al. 2018). In addition, aptamers, a single-stranded DNA or RNA that can specifically bind to cognate molecular targets, can be delivered into cells to specifically blocking *O*-GlcNAcylation on the corresponding sites of the target proteins (Zhu and Chen 2018). Furthermore, nanobodies have also been considered by researchers. Ramirez et al. (Ramirez et al. 2021) reported a nanobody-OGT fusion protein that can selectively increase *O*-GlcNAc levels of target proteins in cells, but does not disrupt the crosstalk or protein structure of PTMs. They also developed a nanobody-fused split OGA for selective removal of *O*-GlcNAc moieties from target proteins in cells and confirmed the effectiveness of the system by testing the target proteins (Ge et al. 2021b). Although there is no clinical report of targeting *O*-GlcNAcylation as a cancer therapy using nanobodies, we have reason to believe that with further research, this technique will show increased potential in the near future.

## Concluding remarks and perspective

In summary, we reviewed the latest literature and silico analyses to show the links between dysregulation of *O*-GlcNAcylation and cancers, and highlighted the great prospects of *O*-GlcNAcylation as a cancer biomarker and therapeutic target. In the past few decades, *O*-GlcNAcylation has gone through a long process from its initial discovery and recognition as an important PTM to being considered as widely involved in all aspects of cellular life activities, even as a central hub of certain metabolic and signal transduction pathways. Although researchers have now realized that protein *O*-GlcNAcylation plays an important role in various human diseases, its detailed mechanisms in these diseases remains to be explored. Especially in cancer, determining how *O*-GlcNAcylation is involved in the regulation of numerous hallmarks of cancer, such as metabolic reprogramming, genomic instability, induction of angiogenesis, changes in the tumor microenvironment, and immune evasion, requires more exhaustive studies.

Hyper-*O*-GlcNAcylation occurs in most cancers, and can affect the growth, proliferation, invasion, metastasis, and chemoresistance in cancer cells. In addition, *O*-GlcNAcylation is involved in multiple signaling pathways and affects the expression of a variety of downstream molecules, such as HIF-1α, c-MYC, AMPK, mTOR, and NF-κB, all of which are main players in oncogenic pathways. Therefore, OGT or *O*-GlcNAcylation can be targeted for cancer therapy, by OGT inhibitors to regulate *O*-GlcNAc levels in cells, with the aim of treating cancer. This appears to be a promising approach, because many OGT inhibitors have shown tumor suppressive effects. However, there are still many obstacles to the entry of OGT inhibitors into clinical practice. First, we still need to study the physiological role of *O*-GlcNAcylation, because rashly changing the global *O*-GlcNAcylation in cells will cause changes in the *O*-GlcNAcylation level of many proteins unrelated to the disease, which might have serious consequences. Second, small molecule inhibitors that target OGT might present off-target toxicity, and most of them are not cell-permeable, thus the development of highly specific and cell-permeable OGT inhibitors is required. In addition, the anticancer effects of existing OGT inhibitors have not been tested in strict animal models and no kinetic and pharmacodynamic data have been reported. In other words, more preclinical data on OGT inhibitors are required. Furthermore, targeting a specific protein modified by *O*-GlcNAcylation is also a potential therapy, and currently emerging technologies, such as the CRISPR-Cas9 system, support the eliminate *O*-GlcNAcylation of proteins; however, these technologies are relatively new and the pathway to put them into clinical practice is long.

## Data Availability

The datasets supporting the conclusions of this article are available in the TIMER database (http://timer.comp-genomics.org), the GEPIA2 database (http://gepia2.cancer-pku.cn), the TCGA database (http://cancergenome.nih.gov), the TISIDB database (http://cis.hku.hk/TISIDB), and the DepMap-portal database (https://depmap.org/portal).

## Abbreviations

*O*-GlcNAc: *O*-Linked β-d-*N*-acetylglucosamine
PTM: Post-translational modification
HBP: Hexosamine biosynthetic pathway
GFAT: Glutamine fructose-6-phosphate amidotransferase
OGT: *O*-GlcNAc transferase
OGA: β-*N*-Acetylglucosaminidase
ncOGT: Nucleocytoplasmic OGT
mOGT: Mitochondrial
sOGT: Short-form OGT
MTS: Mitochondrial targeting sequence
TPR: Tripeptide repeat
NLS: Nuclear localization sequence
AMPK: AMP-activated protein kinase
GSK3β: Glycogen synthase kinase-3β
PGC-1α: Peroxisome proliferator-activated receptor gamma coactivator 1-alpha
FOXO: Forkhead box O1
TCGA: The Cancer Genome Atlas
GEPIA: Gene expression profiling interactive analysis
CHOL: Cholangiocarcinoma
DLBC: Lymphoid neoplasm diffuse large B cell lymphoma
LAML: Acute myeloid leukemia
ACC: Adrenocortical carcinoma
BRCA: Breast invasive carcinoma
CESC: Cervical squamous cell carcinoma and endocervical adenocarcinoma
COAD: Colon adenocarcinoma
LUAD: Lung adenocarcinoma
LUSC: Lung squamous cell carcinoma
OV: Ovarian serous cystadenocarcinoma
PAAD: Pancreatic adenocarcinoma
READ: Rectum adenocarcinoma
SKCM: Skin cutaneous melanoma
THCA: Thyroid carcinoma
UCEC: Uterine corpus endometrial carcinoma
UCS: Uterine carcinosarcoma
TNM: Tumor-node-metastasis
TISIDB: Tumor and Immune System Interaction Database
LIHC: Liver hepatocellular carcinoma
BLCA: Bladder urothelial carcinoma
TGCT: Testicular germ cell tumors
OGA-L: Long form OGA
OGA-S: Short form OGA
GH84: Glycoside hydrolase family 84
HAT: Histone acetyltransferase
PCPG: Pheochromocytoma and paraganglioma
LGG: Brain lower grade glioma
HNSC: Head and neck squamous cell carcinoma
STAD: Stomach adenocarcinoma
MESO: Mesothelioma
OS: Overall survival
BLCA: Bladder urothelial carcinoma
HPV: Human papilloma virus
HCF-1: Host cell factor C1
NF-κB: Nuclear factor kappa B
CXCR4: C-X-C chemokine receptor 4
FOXM1: Forkhead box M1
SKP2: S-phase kinase-associated protein 2
P27^Kip1^: Cyclin-dependent protein kinase inhibitor
SIRT1: Sirtuin 1
ERK: Extracellular regulated kinase
DFS: Disease-free survival
EMT: Epithelial–mesenchymal transition
*WNT5B*: Wnt family member 5B
MLC1: Modulator of VRAC current 1
ROCK: Rho associated coiled-coil containing protein kinase
HCC: Hepatocellular carcinoma
YAP: Yes-associated protein
AEGR: The advanced glycosylation end product-specific receptor
TRIB2: Tribbles pseudokinase 2
HDAC1: Histone deacetylase-1
NAFLD-HCC: Non-alcoholic fatty liver disease-related hepatocellular carcinoma
ER: Endoplasmic reticulum
JNK: JUN N-terminal kinase
c-Jun: AP-1 transcription factor subunit
AP-1: Activator protein 1
LT: Liver transplantation
MMP: Matrix metalloproteinase
AFP: Alpha fetoprotein
RACK1: Ribosome-activated C kinase 1
CRC: Colorectal cancer
YY1: YIN-YANG-1
SLC22A15: Solute carrier family 22 member 15
AANAT: Aralkylamine *N*-acetyltransferase
DDX5: DEAD-box helicase 5
AKT: Protein kinase B
mTOR: Rapamycin kinase
ITGA5: Integrin α5
CCSCs: Colon cancer stem cells
MYBL1: MYB proto-oncogene like 1
PDAC: Pancreatic ductal adenocarcinoma
PD-L1: Programmed cell death 1 ligand 1
HSF1: Heat shock factor 1
HSPs: Heat shock proteins
GC: Gastric cancer
PI3K: Phosphatidylinositol-4,5-bisphosphate 3-kinase
G protein: Guanine nucleotide binding protein
GNB2L1: Beta polypeptide 2-like 1
ESCC: Esophageal squamous cell carcinoma
ESCSs: Esophageal cancer stem cells
PD1: Programmed cell death 1
PC: Prostate cancer
VEGF: Vascular endothelial growth factor
BMI-1: Polycomb ring finger
PRC1: Protein regulator of cytokinesis 1
TP53: Tumor protein 53
PTEN: Phosphatase and tensin homolog
CDKN: Cyclin dependent kinase Inhibitor
BC: Bladder cancer
NUSAP1: Nucleolar and spindle associated protein 1
CDK5: Cyclin-dependent kinase 5
RCC: Renal cell carcinoma
EGFR: Epidermal growth factor receptor
IL-6: Interleukin 6
SCLC: Squamous cell laryngeal cancer
CLL: Chronic lymphocytic leukemia
RSV: Resveratrol
MDS: Myelodysplastic syndrome
AML: Acute myeloid leukemia
ALL: Acute lymphoblastic leukemia
DON: 6-Diazo-5-oxo-l-norleucine
OSMI-1: OGT with a small molecule inhibitor
BADGP: Benzyl-2-acetamido-2-deoxy-α-d-galactopyranoside
DOX: Doxorubicin
CPT: Camptothecin
pre-B-ALL: Pre-B acute lymphoblastic leukemia
ASXL1: Additional sex combs like transcriptional regulator 1
TC: Thyroid cancer
IGF: Insulin-like growth factor
HR: HR lysine demethylase and nuclear receptor corepressor
ACSS2: Acetyl-CoA synthetase 2
Shh: Sonic hedgehog
GNPs: Granule neuron precursors
HER2: Human epidermal growth factor receptor 2
PKM2: Pyruvate kinase isoenzyme M2
HTS: High-throughput screening
ROS: Reactive oxygen species
TRAIL: Tumor necrosis factor (TNF)-related apoptosis inducing ligand
BCL2: B-cell CLL/lymphoma 2
HIF-1α: Hypoxia inducible factor subunit 1 alpha

## References

[CR1] Abdel-Wahab O, Patel J, Levine RL (2011). Clinical implications of novel mutations in epigenetic modifiers in AML. Hematol Oncol Clin North Am.

[CR2] Akella NM, Le Minh G, Ciraku L (2020). *O*-GlcNAc transferase regulates cancer stem-like potential of breast cancer cells. Mol Cancer Res.

[CR3] Akimoto Y, Miura Y, Toda T (2011). Morphological changes in diabetic kidney are associated with increased *O*-GlcNAcylation of cytoskeletal proteins including alpha-actinin 4. Clin Proteomics.

[CR4] Akimov V, Barrio-Hernandez I, Hansen S (2018). UbiSite approach for comprehensive mapping of lysine and N-terminal ubiquitination sites. Nat Struct Mol Biol.

[CR5] Ali A, Kim SH, Kim MJ (2017). *O*-GlcNAcylation of NF-kappaB promotes lung metastasis of cervical cancer cells via upregulation of CXCR4 expression. Mol Cells.

[CR6] Alonso J, Schimpl M, van Aalten DM (2014). *O*-GlcNAcase: promiscuous hexosaminidase or key regulator of *O*-GlcNAc signaling?. J Biol Chem.

[CR7] Arnold CS, Johnson GV, Cole RN (1996). The microtubule-associated protein tau is extensively modified with O-linked *N*-acetylglucosamine. J Biol Chem.

[CR8] Asthana A, Ramakrishnan P, Vicioso Y, Zhang K, Parameswaran R (2018). Hexosamine biosynthetic pathway inhibition leads to AML cell differentiation and cell death. Mol Cancer Ther.

[CR9] Banerjee S, Sangwan V, McGinn O (2013). Triptolide-induced cell death in pancreatic cancer is mediated by *O*-GlcNAc modification of transcription factor Sp1. J Biol Chem.

[CR10] Bejar R, Levine R, Ebert BL (2011). Unraveling the molecular pathophysiology of myelodysplastic syndromes. J Clin Oncol.

[CR11] Bertram L, Blacker D, Mullin K (2000). Evidence for genetic linkage of Alzheimer's disease to chromosome 10q. Science.

[CR12] Blum A, Wang P, Zenklusen JC (2018). SnapShot: TCGA-analyzed tumors. Cell.

[CR13] Bond MR, Hanover JA (2015). A little sugar goes a long way: the cell biology of *O*-GlcNAc. J Cell Biol.

[CR14] Borodkin VS, Schimpl M, Gundogdu M (2014). Bisubstrate UDP-peptide conjugates as human *O*-GlcNAc transferase inhibitors. Biochem J.

[CR15] Bullen JW, Balsbaugh JL, Chanda D (2014). Cross-talk between two essential nutrient-sensitive enzymes: *O*-GlcNAc transferase (OGT) and AMP-activated protein kinase (AMPK). J Biol Chem.

[CR16] Butkinaree C, Cheung WD, Park S (2008). Characterization of beta-*N*-acetylglucosaminidase cleavage by caspase-3 during apoptosis. J Biol Chem.

[CR17] Caldwell SA, Jackson SR, Shahriari KS (2010). Nutrient sensor *O*-GlcNAc transferase regulates breast cancer tumorigenesis through targeting of the oncogenic transcription factor FoxM1. Oncogene.

[CR18] Cancer Cell Line Encyclopedia Consortium, Genomics of Drug Sensitivity in Cancer Consortium (2015). Pharmacogenomic agreement between two cancer cell line data sets. Nature..

[CR19] Cerami E, Gao J, Dogrusoz U (2012). The cBio cancer genomics portal: an open platform for exploring multidimensional cancer genomics data. Cancer Discov.

[CR20] Cervoni L, Turano C, Ferraro A (1997). Glycosylation of RNA polymerase II from wheat germ. Febs Lett.

[CR21] Champattanachai V, Netsirisawan P, Chaiyawat P (2013). Proteomic analysis and abrogated expression of *O*-GlcNAcylated proteins associated with primary breast cancer. Proteomics.

[CR22] Chatham JC, Not LG, Fulop N, Marchase RB (2008). Hexosamine biosynthesis and protein *O*-glycosylation: the first line of defense against stress, ischemia, and trauma. Shock.

[CR23] Chen Y, Liu J, Zhang W (2021). *O*-GlcNAcylation enhances NUSAP1 stability and promotes bladder cancer aggressiveness. Onco Targets Ther.

[CR24] Chen L, Li Y, Song Z (2022). *O*-GlcNAcylation promotes cerebellum development and medulloblastoma oncogenesis via SHH signaling. Proc Natl Acad Sci USA.

[CR25] Cheng S, Ren J, Su L (2016). *O*-GlcNAcylation of the signaling scaffold protein, GNB2L1 promotes its degradation and increases metastasis of gastric tumours. Biochem Biophys Res Commun.

[CR26] Cheng YU, Li H, Li J (2016). *O*-GlcNAcylation enhances anaplastic thyroid carcinoma malignancy. Oncol Lett.

[CR27] Cheung WD, Hart GW (2008). AMP-activated protein kinase and p38 MAPK activate *O*-GlcNAcylation of neuronal proteins during glucose deprivation. J Biol Chem.

[CR28] Ciraku L, Bacigalupa ZA, Ju J (2022). *O*-GlcNAc transferase regulates glioblastoma acetate metabolism via regulation of CDK5-dependent ACSS2 phosphorylation. Oncogene.

[CR29] Clark RJ, McDonough PM, Swanson E (2003). Diabetes and the accompanying hyperglycemia impairs cardiomyocyte calcium cycling through increased nuclear *O*-GlcNAcylation. J Biol Chem.

[CR30] Cohen P (2000). The regulation of protein function by multisite phosphorylation—a 25 year update. Trends Biochem Sci.

[CR31] Davis LI, Blobel G (1987). Nuclear pore complex contains a family of glycoproteins that includes p62: glycosylation through a previously unidentified cellular pathway. Proc Natl Acad Sci USA.

[CR32] de Queiroz RM, Madan R, Chien J, Dias WB, Slawson C (2016). Changes in O-linked *N*-acetylglucosamine (*O*-GlcNAc) homeostasis activate the p53 pathway in ovarian cancer cells. J Biol Chem.

[CR33] Degrell P, Cseh J, Mohas M (2009). Evidence of O-linked *N*-acetylglucosamine in diabetic nephropathy. Life Sci.

[CR34] Dennis JW, Lau KS, Demetriou M, Nabi IR (2009). Adaptive regulation at the cell surface by *N*-glycosylation. Traffic.

[CR35] Dephoure N, Zhou C, Villen J (2008). A quantitative atlas of mitotic phosphorylation. Proc Natl Acad Sci USA.

[CR36] Ding M, Vandre DD (1996). High molecular weight microtubule-associated proteins contain O-linked-*N*-acetylglucosamine. J Biol Chem.

[CR37] Dorfmueller HC, Borodkin VS, Blair DE (2011). Substrate and product analogues as human *O*-GlcNAc transferase inhibitors. Amino Acids.

[CR38] Duan F, Wu H, Jia D (2018). *O*-GlcNAcylation of RACK1 promotes hepatocellular carcinogenesis. J Hepatol.

[CR39] Erratum: Global cancer statistics 2018: GLOBOCAN estimates of incidence and mortality worldwide for 36 cancers in 185 countries. CA Cancer J Clin. 2020;70(4):313. 10.3322/caac.21609 32767693

[CR40] Fan Q, Moen A, Anonsen JH (2018). O-GlcNAc site-mapping of liver X receptor-alpha and *O*-GlcNAc transferase. Biochem Biophys Res Commun.

[CR41] Fardini Y, Dehennaut V, Lefebvre T, Issad T (2013). *O*-GlcNAcylation: a new cancer hallmark?. Front Endocrinol (lausanne).

[CR42] Ferrer CM, Lynch TP, Sodi VL (2014). *O*-GlcNAcylation regulates cancer metabolism and survival stress signaling via regulation of the HIF-1 pathway. Mol Cell.

[CR43] Ferrer CM, Sodi VL, Reginato MJ (2016). *O*-GlcNAcylation in cancer biology: linking metabolism and signaling. J Mol Biol.

[CR44] Ferrer CM, Lu TY, Bacigalupa ZA (2017). *O*-GlcNAcylation regulates breast cancer metastasis via SIRT1 modulation of FOXM1 pathway. Oncogene.

[CR45] Fulop N, Marchase RB, Chatham JC (2007). Role of protein *O*-linked *N*-acetyl-glucosamine in mediating cell function and survival in the cardiovascular system. Cardiovasc Res.

[CR46] Gao Y, Wells L, Comer FI, Parker GJ, Hart GW (2001). Dynamic *O*-glycosylation of nuclear and cytosolic proteins: cloning and characterization of a neutral, cytosolic beta-*N*-acetylglucosaminidase from human brain. J Biol Chem.

[CR47] Ge X, Peng X, Li M (2021). OGT regulated *O*-GlcNacylation promotes migration and invasion by activating IL-6/STAT3 signaling in NSCLC cells. Pathol Res Pract.

[CR48] Ge Y, Ramirez DH, Yang B (2021). Target protein deglycosylation in living cells by a nanobody-fused split *O*-GlcNAcase. Nat Chem Biol.

[CR49] Gelsi-Boyer V, Trouplin V, Adelaide J (2009). Mutations of polycomb-associated gene ASXL1 in myelodysplastic syndromes and chronic myelomonocytic leukaemia. Br J Haematol.

[CR50] Gloster TM, Zandberg WF, Heinonen JE (2011). Hijacking a biosynthetic pathway yields a glycosyltransferase inhibitor within cells. Nat Chem Biol.

[CR51] Golks A, Guerini D (2008). The O-linked *N*-acetylglucosamine modification in cellular signalling and the immune system'. Protein modifications: beyond the usual suspects' review series. Embo Rep.

[CR52] Golks A, Tran TT, Goetschy JF, Guerini D (2007). Requirement for O-linked *N*-acetylglucosaminyltransferase in lymphocytes activation. Embo J.

[CR53] Gong CX, Liu F, Iqbal K (2012). *O*-GlcNAc cycling modulates neurodegeneration. Proc Natl Acad Sci USA.

[CR54] Griffin ME, Jensen EH, Mason DE (2016). Comprehensive mapping of *O*-GlcNAc modification sites using a chemically cleavable tag. Mol Biosyst.

[CR55] Gross BJ, Kraybill BC, Walker S (2005). Discovery of *O*-GlcNAc transferase inhibitors. J Am Chem Soc.

[CR56] Gu Y, Mi W, Ge Y (2010). GlcNAcylation plays an essential role in breast cancer metastasis. Cancer Res.

[CR57] Gu Y, Gao J, Han C (2014). *O*-GlcNAcylation is increased in prostate cancer tissues and enhances malignancy of prostate cancer cells. Mol Med Rep.

[CR58] Guo H, Zhang B, Nairn AV (2017). O-Linked *N*-acetylglucosamine (*O*-GlcNAc) expression levels epigenetically regulate colon cancer tumorigenesis by affecting the cancer stem cell compartment via modulating expression of transcriptional factor MYBL1. J Biol Chem.

[CR59] Gurel B, Iwata T, Koh CM (2008). Nuclear MYC protein overexpression is an early alteration in human prostate carcinogenesis. Mod Pathol.

[CR60] Han SW, Roman J (2006). Fibronectin induces cell proliferation and inhibits apoptosis in human bronchial epithelial cells: pro-oncogenic effects mediated by PI3-kinase and NF-kappa B. Oncogene.

[CR61] Hanahan D, Weinberg RA (2011). Hallmarks of cancer: the next generation. Cell.

[CR62] Hanover JA (2001). Glycan-dependent signaling: O-linked *N*-acetylglucosamine. FASEB J.

[CR63] Hanover JA, Yu S, Lubas WB (2003). Mitochondrial and nucleocytoplasmic isoforms of O-linked GlcNAc transferase encoded by a single mammalian gene. Arch Biochem Biophys.

[CR64] Harosh-Davidovich SB, Khalaila I (2018). *O*-GlcNAcylation affects beta-catenin and E-cadherin expression, cell motility and tumorigenicity of colorectal cancer. Exp Cell Res.

[CR65] Hart GW (2019). Nutrient regulation of signaling and transcription. J Biol Chem.

[CR66] Hawksworth D, Ravindranath L, Chen Y (2010). Overexpression of C-MYC oncogene in prostate cancer predicts biochemical recurrence. Prostate Cancer Prostatic Dis.

[CR67] He N, Ma D, Tan Y, Liu M. Upregulation of *O*-GlcNAc transferase is involved in the pathogenesis of acute myeloid leukemia. Asia Pac J Clin Oncol. 2021. 10.1111/ajco.13685 34821067

[CR68] Housley MP, Rodgers JT, Udeshi ND (2008). *O*-GlcNAc regulates FoxO activation in response to glucose. J Biol Chem.

[CR69] Housley MP, Udeshi ND, Rodgers JT (2009). A PGC-1alpha-*O*-GlcNAc transferase complex regulates FoxO transcription factor activity in response to glucose. J Biol Chem.

[CR70] Hu Y, Suarez J, Fricovsky E (2009). Increased enzymatic *O*-GlcNAcylation of mitochondrial proteins impairs mitochondrial function in cardiac myocytes exposed to high glucose. J Biol Chem.

[CR71] Inoue D, Fujino T, Sheridan P (2018). A novel ASXL1-OGT axis plays roles in H3K4 methylation and tumor suppression in myeloid malignancies. Leukemia.

[CR72] Issad T, Masson E, Pagesy P (2010). *O*-GlcNAc modification, insulin signaling and diabetic complications. Diabetes Metab.

[CR73] Itkonen HM, Minner S, Guldvik IJ (2013). *O*-GlcNAc transferase integrates metabolic pathways to regulate the stability of c-MYC in human prostate cancer cells. Cancer Res.

[CR74] Itkonen HM, Gorad SS, Duveau DY (2016). Inhibition of O-GlcNAc transferase activity reprograms prostate cancer cell metabolism. Oncotarget.

[CR75] Itkonen HM, Urbanucci A, Martin SE (2019). High OGT activity is essential for MYC-driven proliferation of prostate cancer cells. Theranostics.

[CR76] Itkonen HM, Loda M, Mills IG (2021). *O*-GlcNAc transferase—an auxiliary factor or a full-blown oncogene?. Mol Cancer Res.

[CR77] Iyer SP, Akimoto Y, Hart GW (2003). Identification and cloning of a novel family of coiled-coil domain proteins that interact with *O*-GlcNAc transferase. J Biol Chem.

[CR78] Jackson SP, Tjian R (1988). *O*-glycosylation of eukaryotic transcription factors: implications for mechanisms of transcriptional regulation. Cell.

[CR79] Jang TJ, Kim UJ (2016). *O*-GlcNAcylation is associated with the development and progression of gastric carcinoma. Pathol Res Pract.

[CR80] Jaskiewicz NM, Townson DH (2019). Hyper-*O*-GlcNAcylation promotes epithelial–mesenchymal transition in endometrial cancer cells. Oncotarget.

[CR81] Jiang M, Qiu Z, Zhang S (2016). Elevated *O*-GlcNAcylation promotes gastric cancer cells proliferation by modulating cell cycle related proteins and ERK 1/2 signaling. Oncotarget.

[CR82] Jin FZ, Yu C, Zhao DZ, Wu MJ, Yang Z (2013). A correlation between altered *O*-GlcNAcylation, migration and with changes in E-cadherin levels in ovarian cancer cells. Exp Cell Res.

[CR83] Jin L, Lu MH, Dai GC (2020). *O*-GlcNAcylation promotes malignant phenotypes of bladder cancer cells. Neoplasma.

[CR84] Jin L, Yuan F, Dai G (2020). Blockage of O-linked GlcNAcylation induces AMPK-dependent autophagy in bladder cancer cells. Cell Mol Biol Lett.

[CR85] Ju KE (2020). *O*-GlcNAc transferase: structural characteristics, catalytic mechanism and small-molecule inhibitors. ChemBioChem.

[CR86] Kaasik K, Kivimae S, Allen JJ (2013). Glucose sensor O-GlcNAcylation coordinates with phosphorylation to regulate circadian clock. Cell Metab.

[CR87] Kamigaito T, Okaneya T, Kawakubo M (2014). Overexpression of *O*-GlcNAc by prostate cancer cells is significantly associated with poor prognosis of patients. Prostate Cancer Prostatic Dis.

[CR88] Kelly WG, Dahmus ME, Hart GW (1993). RNA polymerase II is a glycoprotein. Modification of the COOH-terminal domain by *O*-GlcNAc. J Biol Chem.

[CR89] Kim M, Kim YS, Kim H (2016). O-linked *N*-acetylglucosamine transferase promotes cervical cancer tumorigenesis through human papillomaviruses E6 and E7 oncogenes. Oncotarget.

[CR90] Konrad RJ, Zhang F, Hale JE (2002). Alloxan is an inhibitor of the enzyme O-linked *N*-acetylglucosamine transferase. Biochem Biophys Res Commun.

[CR91] Kreppel LK, Hart GW (1999). Regulation of a cytosolic and nuclear *O*-GlcNAc transferase. Role of the tetratricopeptide repeats. J Biol Chem.

[CR92] Kreppel LK, Blomberg MA, Hart GW (1997). Dynamic glycosylation of nuclear and cytosolic proteins. Cloning and characterization of a unique *O*-GlcNAc transferase with multiple tetratricopeptide repeats. J Biol Chem.

[CR93] Krzeslak A, Pomorski L, Lipinska A (2010). Elevation of nucleocytoplasmic beta-*N*-acetylglucosaminidase (O-GlcNAcase) activity in thyroid cancers. Int J Mol Med.

[CR94] Krzeslak A, Jozwiak P, Lipinska A (2011). Down-regulation of beta-*N*-acetyl-d-glucosaminidase increases Akt1 activity in thyroid anaplastic cancer cells. Oncol Rep.

[CR95] Krzeslak A, Wojcik-Krowiranda K, Forma E, Bienkiewicz A, Brys M (2012). Expression of genes encoding for enzymes associated with *O*-GlcNAcylation in endometrial carcinomas: clinicopathologic correlations. Ginekol Pol.

[CR96] Kuo WL, Tseng LL, Chang CC et al. Prognostic significance of *O*-GlcNAc and PKM2 in hormone receptor-positive and HER2-nonenriched breast cancer. Diagnostics (Basel). 2021;11(8) 10.3390/diagnostics11081460 PMC8392504 34441396

[CR97] Kuwano R, Miyashita A, Arai H (2006). Dynamin-binding protein gene on chromosome 10q is associated with late-onset Alzheimer's disease. Hum Mol Genet.

[CR98] Laczy B, Hill BG, Wang K (2009). Protein *O*-GlcNAcylation: a new signaling paradigm for the cardiovascular system. Am J Physiol Heart Circ Physiol.

[CR99] Lazarus BD, Love DC, Hanover JA (2009). *O*-GlcNAc cycling: implications for neurodegenerative disorders. Int J Biochem Cell Biol.

[CR100] Lee SJ, Kwon OS. O-GlcNAc transferase inhibitor synergistically enhances doxorubicin-induced apoptosis in HepG2 cells. Cancers (Basel). 2020;12(11). 10.3390/cancers12113154 PMC7693581 33121131

[CR101] Lee SJ, Lee DE, Choi SY, Kwon OS. OSMI-1 Enhances TRAIL-Induced Apoptosis through ER Stress and NF-kappaB Signaling in Colon Cancer Cells. Int J Mol Sci. 2021;22(20). 10.3390/ijms222011073 PMC8539180 34681736

[CR102] Li Y, Wang L, Liu J (2017). *O*-GlcNAcylation modulates Bmi-1 protein stability and potential oncogenic function in prostate cancer. Oncogene.

[CR103] Li T, Fu J, Zeng Z (2020). TIMER2.0 for analysis of tumor-infiltrating immune cells. Nucleic Acids Res.

[CR104] Li X, Wu Z, He J (2021). OGT regulated *O*-GlcNAcylation promotes papillary thyroid cancer malignancy via activating YAP. Oncogene.

[CR105] Li HJ, Wang Y, Li BX (2021). Roles of ten-eleven translocation family proteins and their O-linked beta-*N*-acetylglucosaminylated forms in cancer development. Oncol Lett.

[CR106] Liberti MV, Locasale JW (2016). The Warburg effect: how does it benefit cancer cells?. Trends Biochem Sci.

[CR107] Lin YC, Lin CH, Yeh YC (2018). High O-linked *N*-acetylglucosamine transferase expression predicts poor survival in patients with early stage lung adenocarcinoma. Oncotarget.

[CR108] Liptay S, Weber CK, Ludwig L (2003). Mitogenic and antiapoptotic role of constitutive NF-kappaB/Rel activity in pancreatic cancer. Int J Cancer.

[CR109] Liu Z, Zeng W, Wang S (2017). A potential role for the Hippo pathway protein, YAP, in controlling proliferation, cell cycle progression, and autophagy in BCPAP and KI thyroid papillary carcinoma cells. Am J Transl Res.

[CR110] Liu Y, Cao Y, Pan X (2018). *O*-GlcNAc elevation through activation of the hexosamine biosynthetic pathway enhances cancer cell chemoresistance. Cell Death Dis.

[CR111] Liu L, Li L, Ma C (2019). *O*-GlcNAcylation of Thr(12)/Ser(56) in short-form *O*-GlcNAc transferase (sOGT) regulates its substrate selectivity. J Biol Chem.

[CR112] Liu Z, Zeng W, Maimaiti Y (2019). High expression of yes-activated protein-1 in papillary thyroid carcinoma correlates with poor prognosis. Appl Immunohisto M m.

[CR113] Lode L, Cymbalista F, Soussi T (2016). Genetic profiling of CLL: a 'TP53 addict' perspective. Cell Death Dis.

[CR114] Love DC, Hanover JA (2005). The hexosamine signaling pathway: deciphering the "O-GlcNAc code". Sci STKE.

[CR115] Love DC, Kochan J, Cathey RL, Shin SH, Hanover JA (2003). Mitochondrial and nucleocytoplasmic targeting of O-linked GlcNAc transferase. J Cell Sci.

[CR116] Lubas WA, Frank DW, Krause M, Hanover JA (1997). O-Linked GlcNAc transferase is a conserved nucleocytoplasmic protein containing tetratricopeptide repeats. J Biol Chem.

[CR117] Luthi T, Haltiwanger RS, Greengard P, Bahler M (1991). Synapsins contain O-linked *N*-acetylglucosamine. J Neurochem.

[CR118] Lynch TP, Ferrer CM, Jackson SR (2012). Critical role of O-Linked beta-*N*-acetylglucosamine transferase in prostate cancer invasion, angiogenesis, and metastasis. J Biol Chem.

[CR119] Ma J, Hart GW (2013). Protein *O*-GlcNAcylation in diabetes and diabetic complications. Expert Rev Proteomics.

[CR120] Ma J, Hart GW (2014). O-GlcNAc profiling: from proteins to proteomes. Clin Proteomics.

[CR121] Ma Z, Vosseller K (2013). *O*-GlcNAc in cancer biology. Amino Acids.

[CR122] Ma Z, Vosseller K (2014). Cancer metabolism and elevated *O*-GlcNAc in oncogenic signaling. J Biol Chem.

[CR123] Ma Z, Vocadlo DJ, Vosseller K (2013). Hyper-*O*-GlcNAcylation is anti-apoptotic and maintains constitutive NF-kappaB activity in pancreatic cancer cells. J Biol Chem.

[CR124] Makwana V, Dukie AS, Rudrawar S (2020). Investigating the impact of OGT inhibition on doxorubicin- and docetaxel-induced cytotoxicity in PC-3 and WPMY-1 cells. Int J Toxicol.

[CR125] Marshall S, Bacote V, Traxinger RR (1991). Discovery of a metabolic pathway mediating glucose-induced desensitization of the glucose transport system. Role of hexosamine biosynthesis in the induction of insulin resistance. J Biol Chem.

[CR126] Martin S, Tan ZW, Itkonen HM (2018). Structure-based evolution of low nanomolar *O*-GlcNAc transferase inhibitors. J Am Chem Soc.

[CR127] Mi W, Gu Y, Han C (2011). *O*-GlcNAcylation is a novel regulator of lung and colon cancer malignancy. Biochim Biophys Acta.

[CR128] Mukherjee B, Bhattacharya S, Chakraborty S (2015). Is type 2 diabetes mellitus a predisposal cause for developing hepatocellular carcinoma?. Curr Diabetes Rev.

[CR129] Ngoh GA, Facundo HT, Hamid T (2009). Unique hexosaminidase reduces metabolic survival signal and sensitizes cardiac myocytes to hypoxia/reoxygenation injury. Circ Res.

[CR130] Niu Y, Xia Y, Wang J, Shi X (2017). *O*-GlcNAcylation promotes migration and invasion in human ovarian cancer cells via the RhoA/ROCK/MLC pathway. Mol Med Rep.

[CR131] Nolte D, Muller U (2002). Human *O*-GlcNAc transferase (OGT): genomic structure, analysis of splice variants, fine mapping in Xq13.1. Mamm Genome.

[CR132] Olivier-Van SS, Guinez C, Mir AM (2012). The hexosamine biosynthetic pathway and *O*-GlcNAcylation drive the expression of beta-catenin and cell proliferation. Am J Physiol Endocrinol Metab.

[CR133] Olivier-Van SS, Dehennaut V, Buzy A (2014). *O*-GlcNAcylation stabilizes beta-catenin through direct competition with phosphorylation at threonine 41. Faseb J.

[CR134] Olsen JV, Vermeulen M, Santamaria A (2010). Quantitative phosphoproteomics reveals widespread full phosphorylation site occupancy during mitosis. Sci Signal..

[CR135] Ortiz-Meoz RF, Jiang J, Lazarus MB (2015). A small molecule that inhibits OGT activity in cells. Acs Chem Biol.

[CR136] Peng K, Liu R, Jia C et al. Regulation of O-Linked *N*-acetyl glucosamine transferase (OGT) through E6 stimulation of the ubiquitin ligase activity of E6AP. Int J Mol Sci. 2021;22(19) 10.3390/ijms221910286 PMC8508608 34638625

[CR137] Peterson SB, Hart GW (2016). New insights: a role for *O*-GlcNAcylation in diabetic complications. Crit Rev Biochem Mol Biol.

[CR138] Pham LV, Bryant JL, Mendez R (2016). Targeting the hexosamine biosynthetic pathway and O-linked *N*-acetylglucosamine cycling for therapeutic and imaging capabilities in diffuse large B-cell lymphoma. Oncotarget.

[CR139] Phoomak C, Silsirivanit A, Wongkham C (2012). Overexpression of *O*-GlcNAc-transferase associates with aggressiveness of mass-forming cholangiocarcinoma. Asian Pac J Cancer Prev.

[CR140] Phueaouan T, Chaiyawat P, Netsirisawan P (2013). Aberrant *O*-GlcNAc-modified proteins expressed in primary colorectal cancer. Oncol Rep.

[CR141] Pietila M, Ivaska J, Mani SA (2016). Whom to blame for metastasis, the epithelial-mesenchymal transition or the tumor microenvironment?. Cancer Lett.

[CR142] Qian K, Wang S, Fu M (2018). Transcriptional regulation of *O*-GlcNAc homeostasis is disrupted in pancreatic cancer. J Biol Chem.

[CR143] Qiao Z, Dang C, Zhou B (2012). *O*-linked *N*-acetylglucosamine transferase (OGT) is overexpressed and promotes O-linked protein glycosylation in esophageal squamous cell carcinoma. J Biomed Res.

[CR144] Qiao Y, Zhang X, Zhang Y (2016). High glucose stimulates tumorigenesis in hepatocellular carcinoma cells through AGER-dependent *O*-GlcNAcylation of c-Jun. Diabetes.

[CR145] Qiao Z, Dang C, Zhou B (2016). Downregulation of O-linked *N*-acetylglucosamine transferase by RNA interference decreases MMP9 expression in human esophageal cancer cells. Oncol Lett.

[CR146] Ramirez DH, Ge Y, Woo CM (2021). *O*-GlcNAc engineering on a target protein in cells with nanobody-OGT and nanobody-splitOGA. Curr Protoc.

[CR147] Reiter RJ, Rosales-Corral SA, Tan DX et al. Melatonin, a full service anti-cancer agent: inhibition of initiation, progression and metastasis. Int J Mol Sci. 2017;18(4) 10.3390/ijms18040843 PMC5412427 28420185

[CR148] Roman A, Munger K (2013). The papillomavirus E7 proteins. Virology.

[CR149] Roquemore EP, Chevrier MR, Cotter RJ, Hart GW (1996). Dynamic *O*-GlcNAcylation of the small heat shock protein alpha B-crystallin. Biochemistry-Us.

[CR150] Roychowdhury S, Chinnaiyan AM (2016). Translating cancer genomes and transcriptomes for precision oncology. CA Cancer J Clin.

[CR151] Rozanski W, Krzeslak A, Forma E (2012). Prediction of bladder cancer based on urinary content of MGEA5 and OGT mRNA level. Clin Lab.

[CR152] Ru B, Wong CN, Tong Y (2019). TISIDB: an integrated repository portal for tumor-immune system interactions. Bioinformatics.

[CR153] Ryu IH, Do SI (2011). Denitrosylation of *S*-nitrosylated OGT is triggered in LPS-stimulated innate immune response. Biochem Biophys Res Commun.

[CR154] Scheffner M, Werness BA, Huibregtse JM, Levine AJ, Howley PM (1990). The E6 oncoprotein encoded by human papillomavirus types 16 and 18 promotes the degradation of p53. Cell.

[CR155] Schultz J, Pils B (2002). Prediction of structure and functional residues for *O*-GlcNAcase, a divergent homologue of acetyltransferases. Febs Lett.

[CR156] Seo HG, Kim HB, Kang MJ (2016). Identification of the nuclear localisation signal of *O*-GlcNAc transferase and its nuclear import regulation. Sci Rep.

[CR157] Seo HG, Kim HB, Yoon JY (2020). Mutual regulation between OGT and XIAP to control colon cancer cell growth and invasion. Cell Death Dis.

[CR158] Shang M, Yang H, Yang R (2021). The folate cycle enzyme MTHFD2 induces cancer immune evasion through PD-L1 up-regulation. Nat Commun.

[CR159] Shi Y, Tomic J, Wen F (2010). Aberrant *O*-GlcNAcylation characterizes chronic lymphocytic leukemia. Leukemia.

[CR160] Slawson C, Hart GW (2011). *O*-GlcNAc signalling: implications for cancer cell biology. Nat Rev Cancer.

[CR161] Sodi VL, Khaku S, Krutilina R (2015). mTOR/MYC axis regulates *O*-GlcNAc transferase expression and *O*-GlcNAcylation in breast cancer. Mol Cancer Res.

[CR162] Spaner DE (2021). *O*-GlcNAcylation in chronic lymphocytic leukemia and other blood cancers. Front Immunol.

[CR163] Starska K, Forma E, Brzezinska-Blaszczyk E (2015). Gene and protein expression of *O*-GlcNAc-cycling enzymes in human laryngeal cancer. Clin Exp Med.

[CR164] Steenackers A, Olivier-Van SS, Baldini SF (2016). Silencing the nucleocytoplasmic *O*-GlcNAc transferase reduces proliferation, adhesion, and migration of cancer and fetal human colon cell lines. Front Endocrinol (lausanne).

[CR165] Tai HC, Khidekel N, Ficarro SB, Peters EC, Hsieh-Wilson LC (2004). Parallel identification of *O*-GlcNAc-modified proteins from cell lysates. J Am Chem Soc.

[CR166] Takahashi M, Tsujioka Y, Yamada T (1999). Glycosylation of microtubule-associated protein tau in Alzheimer's disease brain. Acta Neuropathol.

[CR167] Tang Z, Kang B, Li C, Chen T, Zhang Z (2019). GEPIA2: an enhanced web server for large-scale expression profiling and interactive analysis. Nucleic Acids Res.

[CR168] Toleman C, Paterson AJ, Whisenhunt TR, Kudlow JE (2004). Characterization of the histone acetyltransferase (HAT) domain of a bifunctional protein with activable *O*-GlcNAcase and HAT activities. J Biol Chem.

[CR169] Tomic J, McCaw L, Li Y (2013). Resveratrol has anti-leukemic activity associated with decreased *O*-GlcNAcylated proteins. Exp Hematol.

[CR170] Torres CR, Hart GW (1984). Topography and polypeptide distribution of terminal *N*-acetylglucosamine residues on the surfaces of intact lymphocytes. Evidence for *O*-linked GlcNAc. J Biol Chem.

[CR171] UniProt Consortium (2019). UniProt: a worldwide hub of protein knowledge. Nucleic Acids Res..

[CR172] Vander HM, Cantley LC, Thompson CB (2009). Understanding the Warburg effect: the metabolic requirements of cell proliferation. Science.

[CR173] Vocadlo DJ (2012). *O*-GlcNAc processing enzymes: catalytic mechanisms, substrate specificity, and enzyme regulation. Curr Opin Chem Biol.

[CR174] Wang W, Abbruzzese JL, Evans DB (1999). The nuclear factor-kappa B RelA transcription factor is constitutively activated in human pancreatic adenocarcinoma cells. Clin Cancer Res.

[CR175] Wang L, Chen S, Zhang Z (2018). Suppressed OGT expression inhibits cell proliferation while inducing cell apoptosis in bladder cancer. BMC Cancer.

[CR176] Wang L, Chen S, Zhang J (2019). Suppressed OGT expression inhibits cell proliferation and modulates EGFR expression in renal cell carcinoma. Cancer Manag Res.

[CR177] Wang J, Wang Z, Yuan J, Wang J, Shen X (2020). The positive feedback between ACSL4 expression and *O*-GlcNAcylation contributes to the growth and survival of hepatocellular carcinoma. Aging (albany NY).

[CR178] Weinstein JN, Collisson EA, Mills GB (2013). The cancer genome atlas pan-cancer analysis project. Nat Genet.

[CR179] Wells L, Vosseller K, Hart GW (2001). Glycosylation of nucleocytoplasmic proteins: signal transduction and *O*-GlcNAc. Science.

[CR180] Woo CM, Lund PJ, Huang AC (2018). Mapping and quantification of over 2000 O-linked glycopeptides in activated human T cells with isotope-targeted glycoproteomics (Isotag). Mol Cell Proteomics.

[CR181] Wu N, Jiang M, Han Y (2019). *O*-GlcNAcylation promotes colorectal cancer progression by regulating protein stability and potential catcinogenic function of DDX5. J Cell Mol Med.

[CR182] Wu J, Tan Z, Li H (2021). Melatonin reduces proliferation and promotes apoptosis of bladder cancer cells by suppressing *O*-GlcNAcylation of cyclin-dependent-like kinase 5. J Pineal Res.

[CR183] Wu D, Jin J, Qiu Z, Liu D, Luo H. Functional analysis of *O*-GlcNAcylation in cancer metastasis. Front Oncol. 2020;10. 10.3389/fonc.2020.585288 PMC7653022 33194731

[CR184] Wulff-Fuentes E, Berendt RR, Massman L (2021). The human *O*-GlcNAcome database and meta-analysis. Sci Data.

[CR185] Xu W, Zhang X, Wu JL (2017). *O*-GlcNAc transferase promotes fatty liver-associated liver cancer through inducing palmitic acid and activating endoplasmic reticulum stress. J Hepatol.

[CR186] Xu Z, Zhang Y, Ocansey D, Wang B, Mao F (2021). Glycosylation in cervical cancer: new insights and clinical implications. Front Oncol.

[CR187] Yang X, Qian K (2017). Protein *O*-GlcNAcylation: emerging mechanisms and functions. Nat Rev Mol Cell Biol.

[CR188] Yang X, Su K, Roos MD (2001). O-linkage of *N*-acetylglucosamine to Sp1 activation domain inhibits its transcriptional capability. Proc Natl Acad Sci USA.

[CR189] Yang WH, Kim JE, Nam HW (2006). Modification of p53 with O-linked *N*-acetylglucosamine regulates p53 activity and stability. Nat Cell Biol.

[CR190] Yao B, Xu Y, Wang J (2016). Reciprocal regulation between *O*-GlcNAcylation and tribbles pseudokinase 2 (TRIB2) maintains transformative phenotypes in liver cancer cells. Cell Signal.

[CR191] Yoshida K, Sanada M, Shiraishi Y (2011). Frequent pathway mutations of splicing machinery in myelodysplasia. Nature.

[CR192] Yu M, Chu S, Fei B, Fang X, Liu Z (2019). *O*-GlcNAcylation of ITGA5 facilitates the occurrence and development of colorectal cancer. Exp Cell Res.

[CR193] Yuan Y, Wang L, Ge D (2021). Exosomal O-GlcNAc transferase from esophageal carcinoma stem cell promotes cancer immunosuppression through up-regulation of PD-1 in CD8(+) T cells. Cancer Lett.

[CR194] Zachara NE, Hart GW (2006). Cell signaling, the essential role of *O*-GlcNAc!. Biochim Biophys Acta.

[CR195] Zeng Q, Zhao RX, Chen J (2016). O-linked GlcNAcylation elevated by HPV E6 mediates viral oncogenesis. Proc Natl Acad Sci USA.

[CR196] Zhang N, Chen X (2016). Potential role of *O*-GlcNAcylation and involvement of PI3K/Akt1 pathway in the expression of oncogenic phenotypes of gastric cancer cells in vitro. Biotechnol Appl Biochem.

[CR197] Zhang H, Gao G, Brunk UT (1992). Extracellular reduction of alloxan results in oxygen radical-mediated attack on plasma and lysosomal membranes. APMIS.

[CR198] Zhang P, Wang C, Ma T, You S (2015). *O*-GlcNAcylation enhances the invasion of thyroid anaplastic cancer cells partially by PI3K/Akt1 pathway. Onco Targets Ther.

[CR199] Zhang B, Zhou P, Li X (2017). Bitterness in sugar: *O*-GlcNAcylation aggravates pre-B acute lymphocytic leukemia through glycolysis via the PI3K/Akt/c-Myc pathway. Am J Cancer Res.

[CR200] Zhang X, Qiao Y, Wu Q (2017). The essential role of YAP O-GlcNAcylation in high-glucose-stimulated liver tumorigenesis. Nat Commun.

[CR201] Zheng SS, Xu X, Wu J (2008). Liver transplantation for hepatocellular carcinoma: Hangzhou experiences. Transplantation.

[CR202] Zhou F, Yang X, Zhao H (2018). Down-regulation of OGT promotes cisplatin resistance by inducing autophagy in ovarian cancer. Theranostics.

[CR203] Zhu G, Chen X (2018). Aptamer-based targeted therapy. Adv Drug Deliv Rev.

[CR204] Zhu Q, Zhou L, Yang Z (2012). *O*-GlcNAcylation plays a role in tumor recurrence of hepatocellular carcinoma following liver transplantation. Med Oncol.

[CR205] Zhu Y, Shan X, Yuzwa SA, Vocadlo DJ (2014). The emerging link between *O*-GlcNAc and Alzheimer disease. J Biol Chem.

[CR206] Zhu G, Tao T, Zhang D (2016). *O*-GlcNAcylation of histone deacetylases 1 in hepatocellular carcinoma promotes cancer progression. Glycobiology.

[CR207] Zhu G, Qian M, Lu L et al. O-GlcNAcylation of YY1 stimulates tumorigenesis in colorectal cancer cells by targeting SLC22A15 and AANAT. Carcinogenesis. 2019. 10.1093/carcin/bgz010 30715269

